# An AAV-based, room-temperature-stable, single-dose COVID-19 vaccine provides durable immunogenicity and protection in non-human primates

**DOI:** 10.1016/j.chom.2021.08.002

**Published:** 2021-09-08

**Authors:** Nerea Zabaleta, Wenlong Dai, Urja Bhatt, Cécile Hérate, Pauline Maisonnasse, Jessica A. Chichester, Julio Sanmiguel, Reynette Estelien, Kristofer T. Michalson, Cheikh Diop, Dawid Maciorowski, Nathalie Dereuddre-Bosquet, Mariangela Cavarelli, Anne-Sophie Gallouët, Thibaut Naninck, Nidhal Kahlaoui, Julien Lemaitre, Wenbin Qi, Elissa Hudspeth, Allison Cucalon, Cecilia D. Dyer, M. Betina Pampena, James J. Knox, Regina C. LaRocque, Richelle C. Charles, Dan Li, Maya Kim, Abigail Sheridan, Nadia Storm, Rebecca I. Johnson, Jared Feldman, Blake M. Hauser, Vanessa Contreras, Romain Marlin, Raphaël Ho Tsong Fang, Catherine Chapon, Sylvie van der Werf, Eric Zinn, Aisling Ryan, Dione T. Kobayashi, Ruchi Chauhan, Marion McGlynn, Edward T. Ryan, Aaron G. Schmidt, Brian Price, Anna Honko, Anthony Griffiths, Sam Yaghmour, Robert Hodge, Michael R. Betts, Mason W. Freeman, James M. Wilson, Roger Le Grand, Luk H. Vandenberghe

**Affiliations:** 1 Grousbeck Gene Therapy Center, Schepens Eye Research Institute, Mass Eye and Ear, Boston, MA, USA; 2 Ocular Genomics Institute, Department of Ophthalmology, Harvard Medical School, Boston, MA, USA; 3 The Broad Institute of Harvard and MIT, Cambridge, MA, USA; 4 Harvard Stem Cell Institute, Harvard University, Cambridge, MA, USA; 5 Center for Immunology of Viral, Auto-immune, Hematological and Bacterial diseases (IMVA-HB/IDMIT), Université Paris-Saclay, Inserm, CEA, Fontenay-aux-Roses, France; 6 Gene Therapy Program, Perelman School of Medicine, University of Pennsylvania, Philadelphia, PA, USA; 7 Novartis Gene Therapies, San Diego, CA, USA; 8 Novartis Gene Therapies, NC, USA; 9 Department of Microbiology, Perelman School of Medicine, University of Pennsylvania, Philadelphia, PA, USA; 10 Department of Pathology, Perelman School of Medicine, University of Pennsylvania, Philadelphia, PA, USA; 11 Division of Infectious Diseases, Massachusetts General Hospital, Boston, MA, USA; 12 Department of Medicine, Harvard Medical School, Boston, MA, USA; 13 Department of Microbiology and National Emerging Infectious Diseases Laboratories, Boston University School of Medicine, Boston, MA 02118, USA; 14 Ragon Institute of MGH, MIT, and Harvard, Cambridge, MA 02139, USA; 15 Molecular Genetics of RNA Viruses, Department of Virology, Institut Pasteur, CNRS UMR 3569, Université de Paris, Paris, France; 16 National Reference Center for Respiratory Viruses, Institut Pasteur, Paris, France; 17 Translational Innovation Fund, Mass General Brigham Innovation, Cambridge, MA, USA; 18 Department of Immunology and Infectious Diseases, Harvard T. H. Chan School of Public Health, Boston, MA, USA; 19 Department of Microbiology, Harvard Medical School, Boston, MA, USA; 20 Albamunity, Boston, MA, USA; 21 Novartis Gene Therapies, Libertyville, IL, USA; 22 Center for Computational & Integrative Biology, Department of Medicine, and Translational Research Center, Massachusetts General Hospital, Harvard Medical School, Boston, MA, USA

**Keywords:** adeno-associated virus, AAV, SARS-CoV-2, COVID-19, vaccine, immunization, single dose, room-temperature stable, viral challenge, durability

## Abstract

The SARS-CoV-2 pandemic has affected more than 185 million people worldwide resulting in over 4 million deaths. To contain the pandemic, there is a continued need for safe vaccines that provide durable protection at low and scalable doses and can be deployed easily. Here, AAVCOVID-1, an adeno-associated viral (AAV), spike-gene-based vaccine candidate demonstrates potent immunogenicity in mouse and non-human primates following a single injection and confers complete protection from SARS-CoV-2 challenge in macaques. Peak neutralizing antibody titers are sustained at 1 year and complemented by functional memory T cell responses. The AAVCOVID vector has no relevant pre-existing immunity in humans and does not elicit cross-reactivity to common AAVs used in gene therapy. Vector genome persistence and expression wanes following injection. The single low-dose requirement, high-yield manufacturability, and 1-month stability for storage at room temperature may make this technology well suited to support effective immunization campaigns for emerging pathogens on a global scale.

## Highlights

• AAVCOVID is a gene- and AAV-based spike antigen COVID-19 vaccine
• A single AAVCOVID dose provides protection from SARS-CoV-2 challenge in monkeys
• Neutralizing antibody levels in animals are maintained at peak for a year
• AAVCOVID can be produced at scale and stored at room temperature for 1 month

## Graphical abstract

**Figure:**
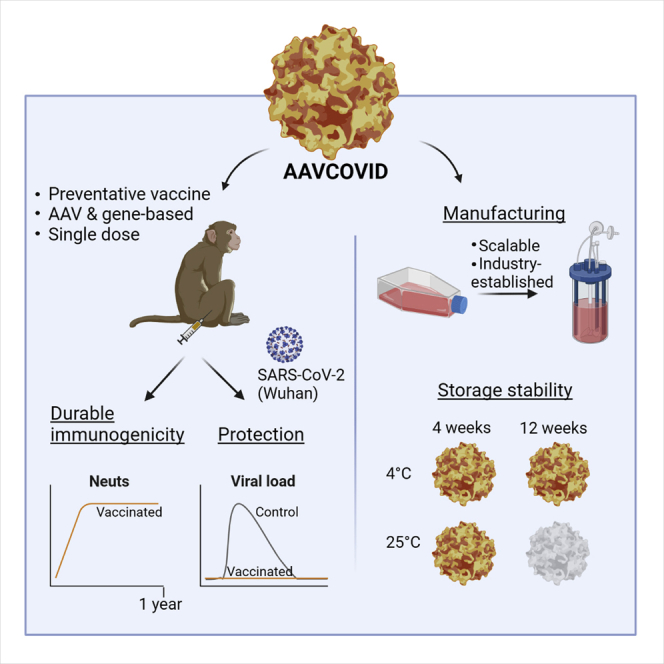

## Teaser

Zabaleta et al. report AAVCOVID, an experimental preventative vaccine for COVID-19. AAVCOVID is a vaccine that delivers the SARS-CoV-2 spike antigen as a gene via an adeno-associated vector. Results demonstrate protection from viral challenge in NHPs, durable immunogenicity, cross-reactivity with emerging variants, a scalable production method, and room-temperature stability for 1 month.

## Introduction

A severe acute respiratory disease syndrome caused by a novel coronavirus was first reported in December 2019 (COVID-19 disease) and was subsequently shown to be caused by SARS-CoV-2 (Zhou et al., 2020). Since early 2020, when the novel pathogen was first isolated and sequenced, dozens of vaccine efforts have been initiated to converge on multiple candidates worldwide. To date, several of these have been approved on the basis of a highly favorable safety and efficacy profile (Baden et al., 2021; Dagan et al., 2021; Polack et al., 2020; Sadoff et al., 2021; Voysey et al., 2021). Based on prior work on SARS-CoV-1 and other respiratory viruses, the SARS-CoV-2 spike protein (S) was considered an attractive antigen target for the induction of protective immunity to the virus (Folegatti et al., 2020a; Graham et al., 2020; Koch et al., 2020; Martin et al., 2008; Modjarrad et al., 2019; Muthumani et al., 2015; van Doremalen et al., 2020a). This viral envelope glycoprotein engages the ACE-2 cellular receptor through its receptor-binding domain (RBD), a key target for developing neutralizing antibodies (Wang et al., 2020). Antigenicity of a full-length S protein can be further enhanced by select proline substitutions that maintain the S protein in a pre-fusion conformation (Walls et al., 2020; Wrapp et al., 2020). The utility of employing this antigen as a vaccination target has been validated by reports of substantial protective efficacy in several human vaccine studies (Folegatti et al., 2020b; Jackson et al., 2020).

The magnitude of this crisis has motivated the initiation of over 200 SARS-CoV-2 vaccines in development across various technology platforms (https://www.who.int/publications/m/item/draft-landscape-of-covid-19-candidate-vaccines). Inactivated viral and gene-based vaccines progressed particularly rapidly with the start of the first phase-1 studies occurring about 3 months after the identification and sequencing of SARS-CoV-2. The various gene-based vaccines encode for SARS-CoV-2 Spike or RBD-containing sequences and leverage different gene delivery platforms including unencapsulated or naked DNA delivered by electroporation (Patel et al., 2020; Yu et al., 2020), mRNA delivered mostly by lipid nanoparticles (Anderson et al., 2020; Corbett et al., 2020; Jackson et al., 2020; Kalnin et al., 2020; Kremsner et al., 2020; Walsh et al., 2020; Zhang et al., 2020), and viral vectors such as vesicular stomatitis virus (VSV) (Case et al., 2020), adenovirus (Feng et al., 2020; Folegatti et al., 2020b; Hassan et al., 2020; Logunov et al., 2020; Mercado et al., 2020; van Doremalen et al., 2020b; Zhu et al., 2020a, 2020b), or yellow fever virus (Sanchez-Felipe et al., 2021). 1 and a half years after the start of the outbreak, at least four of the gene-based vaccine candidates are efficacious and safe in large phase-3 studies and have met requirements for emergency use approval (EUA) by the US Food and Drug Organization and/or the European Medicines Agency (Baden et al., 2021; Dagan et al., 2021; Polack et al., 2020; Sadoff et al., 2021; Voysey et al., 2021).

As public health programs race to vaccinate individuals with these early wave vaccines, some of the limitations of the first-generation vaccines are becoming increasingly apparent, especially for distribution in low- and middle-income countries. The cold-chain storage requirement and reliance on more than one injection to induce protective immunity are major limitations of some of the first-generation vaccines (Folegatti et al., 2020b; Jackson et al., 2020). It is less clear at this time, but issues such as post-inoculation reactogenicity, durability of immune responses, and efficacy in populations with known vulnerabilities to COVID-19, such as the elderly and obese, may also be attributes upon which second wave vaccines can improve (Biswas et al., 2020; Cuschieri and Grech, 2020). Moreover, little is known at this time about the expense and reliability of scale-up manufacturing that will be critical in assessing the feasibility of using any vaccine in global vaccine campaigns. Additionally, new SARS-CoV-2 strains are emerging, and the immune escape of new variants is of concern (Garcia-Beltran et al., 2021; Wu et al., 2021b). Efficacy of re-vaccination against the emerging variants has also been explored (Alter et al., 2021; Collier et al., 2021; Liu et al., 2021; Wu et al., 2021a). Viral vector vaccines that elicit an immune response against the vector might show reduced efficacy after several doses due to the neutralization of the vector.

Here, we interrogate the safety and immunogenicity of a class of experimental vaccines that has previously not been proposed for prevention of COVID-19: the adeno-associated viral vector (AAV). AAV vaccines, not to be confused with adenoviral vaccines, have previously been shown to induce durable, potent humoral and cellular immunogenicity following a single-dose intramuscular injection in mice and non-human primates to various viral pathogens (Li et al., 2012; Lin et al., 2009; Xin et al., 2006; Zhu et al., 2019) and were explored in early stage human studies for HIV in the past (Mehendale et al., 2008; Vardas et al., 2010). Moreover, the AAVrh32.33 platform, on which the COVID-19 vaccine candidates here are build, has no relevant anti-vector pre-existing immunity in humans (Calcedo et al., 2009) and unlike typical AAVs, when encoding a foreign antigen, lead to waning transgene persistence and expression (Lin et al., 2009; Mays et al., 2009, 2013).

Wild-type AAV is a non-enveloped single-stranded DNA dependoparvovirus with broad host range including non-human and human primates. AAV is not known to cause disease; however, antibodies directed at many AAV serotypes are prevalent, suggesting that it is endemic in human populations (Calcedo et al., 2009; Gao et al., 2004). Moreover, AAV vectors do not retain any viral open-reading frames and as such are replication defective. The AAV capsid is known to be a primary determinant of biodistribution, immunogenicity, production yield, and efficiency of gene transfer (Salganik et al., 2015; Vandenberghe et al., 2009). Furthermore, the icosahedral capsid structure of AAV is known to be highly thermostable and thus possibly have reduced cold-chain requirements as a vaccine (Bennett et al., 2017; Pacouret et al., 2017; Rayaprolu et al., 2013). AAV production can be done at scale through a number of manufacturing methods that are well established in the industry to produce dozens of products currently in clinical development and available commercially (Joshi et al., 2020; Robert et al., 2017).

Four decades of preclinical and 25 years of clinical experience with AAV products in gene therapy has established an overall favorable safety profile (High and Roncarolo, 2019). Today, 3 products have been approved by either US FDA or the EMA (Bennett et al., 2016; Mendell et al., 2017; Stroes et al., 2008). AAV is generally well tolerated via several local routes of administration, including intramuscular (IM) injection, and large doses delivered systemically, typically by intravenous injection, have been commonly used in human clinical trials (Bennett et al., 2016; High and Roncarolo, 2019; Mendell et al., 2017; Stroes et al., 2008). The low pro-inflammatory responses to the AAV capsid are known to allow for a tolerogenic or anergic environment to be established toward a self-transgene product (Mingozzi and High, 2017). However, transgenes that are foreign to the host often lead to antigen-specific humoral and cellular immunogenicity, tissue inflammation, and the potential elimination of the transgene product or the transgene-expressing cell (Gao et al., 2009; Vandenberghe and Wilson, 2007). While undesirable in therapeutic gene therapy applications, the inflammatory potential of AAV has been leveraged in several preclinical and clinical vaccine studies (Lin et al., 2009; Mehendale et al., 2008; Nieto and Salvetti, 2014; Vardas et al., 2010).

**Figure 1:**
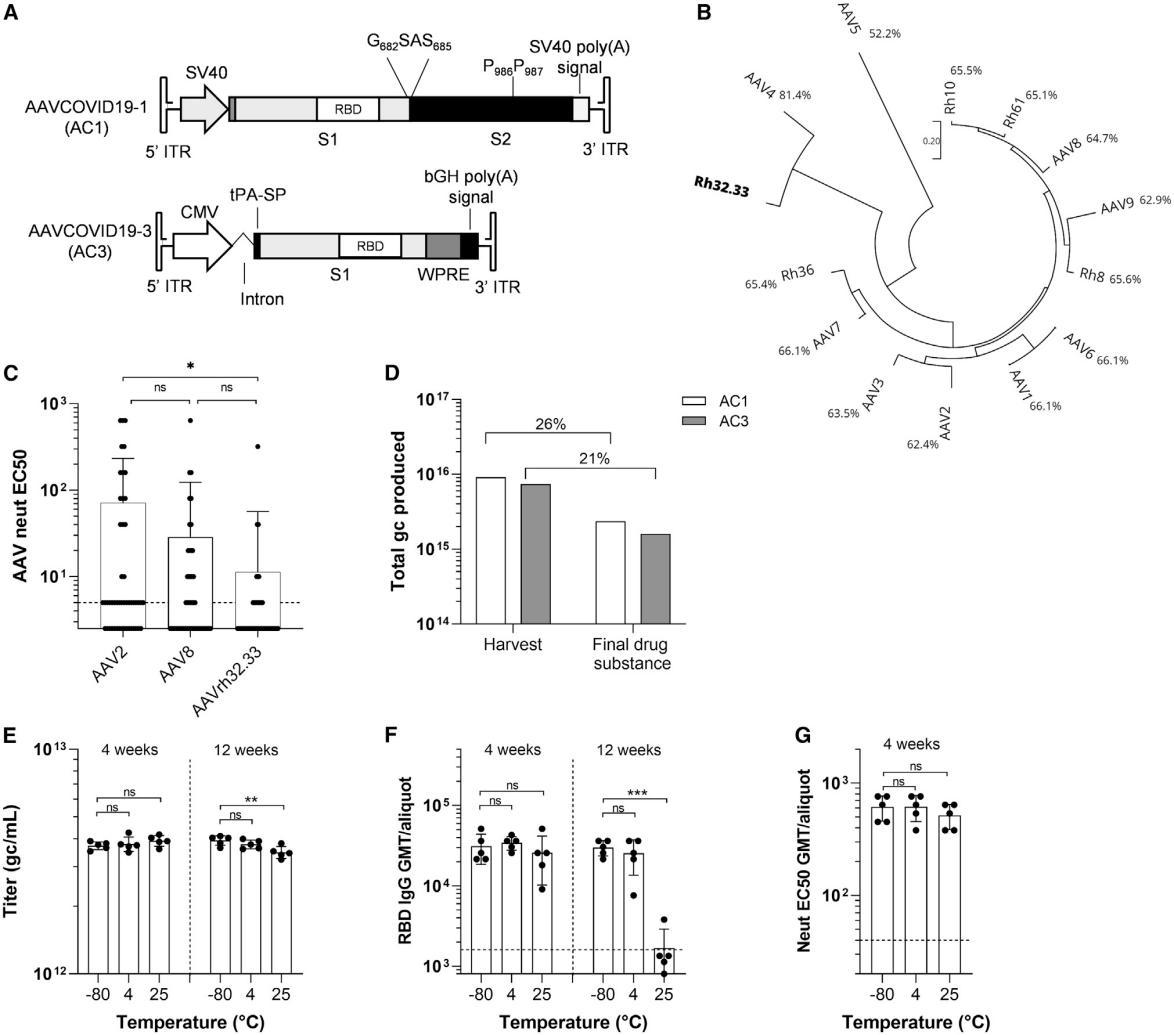
Composition, productivity, and stability of AAVCOVID vaccine candidates (A) Schematic representation of the recombinant genome of AAVCOVID19-1 (AC1) and AAVCOVID19-3 (AC3) vaccine candidates. SV40: Simian virus 40 promoter. RBD: receptor-binding domain. S1, S2: SARS-CoV-2 spike subunits 1 and 2. CMV: cytomegalovirus promoter. tPA-SP, tissue plasminogen activator signal peptide; WPRE, woodchuck hepatitis virus posttranscriptional regulatory element; bGH, bovine growth hormone; ITR, inverted terminal repeat. (B) Phylogenetic tree of several AAV clades and percentage of sequence identity with AAVrh32.33. (C) AAV2, AAV8, and AAVrh32.33 neutralizing antibody titer (EC_50_) in 50 human donor plasma samples. (D) Total genome copies (gc) of AC1 and AC3 produced at large scale and quantified at harvest of producer cells and after the purification (final drug substance). Percentage of vector recovery during purification is displayed. (E) Titer (gc/mL) of AC1 aliquots (n = 5) stored at −80°C, 4°C, and 25°C for 4 and 12 weeks. (F) RBD-binding antibody titers in C57BL/6 animals 21 days after vaccination with 10^11^gc of AC1 aliquots (n = 5) stored at −80°C, 4°C, and 25°C for 4 and 12 weeks. (G) Pseudovirus neutralizing titers (EC_50_) in C57BL/6 animals 21 days after vaccination with 10^11^gc of AC1 aliquots (n = 5) stored at −80°C, 4°C, and 25°C for 4 weeks. (C and E–G) Data are represented as mean ± SD. One-way ANOVA and Tukey’s post-test. ^∗^p < 0.05, ^∗∗^p < 0.01, ^∗∗∗^p < 0.001 See also Figure S1.

AAVrh32.33 is a hybrid serotype capsid that is phylogenetically distinct from commonly used gene therapy AAVs such as AAV2, AAV8, or AAV9, with a sequence identity less than 70% (Figure 1B) (Calcedo et al., 2009; Lin et al., 2009). In contrast to most AAVs studied to date, AAVrh32.33, following intramuscular injection (IM), elicits a pro-inflammatory response in mice, declining transgene expression over time, and pronounced local inflammatory infiltrates (Lin et al., 2009; Mays et al., 2009, 2013). Moreover, in an extensive sero-epidemiological study of AAVrh32.33, less than 2% of subjects carried neutralizing responses above a titer of 1:20 (Calcedo et al., 2009). These rare, low titer antibody levels did not reduce vaccine efficacy due to neutralization when AAVrh32.33 was used as the vaccine vector in contrast to what was observed when other AAV vectors were used to which higher titer antibodies were prevalent (Lin et al., 2009). Previously, these key attributes of AAVrh32.33 compelled us and others to explore the potential of this AAV serotype as a vaccine platform. This earlier work established viability as potential vaccine via proof-of-concept studies in an influenza murine challenge model, a mouse and NHP HIV immunogenicity study, as well as in applications in dengue and HCV infections (Li et al., 2012; Lin et al., 2009; Zhu et al., 2015, 2019). Collectively, these studies provided evidence of potent, durable, and functional antibody and T cell responses.

Here, we hypothesized that an AAVrh32.33 expressing the SARS-CoV-2 S protein can induce protective and durable immunity from a single-dose intramuscular administration. Based on the thermostability properties of AAV, we further sought to interrogate the cold-chain requirements of the AAVCOVID vaccine candidate.

## Results

### Design, production, and stability of AAVCOVID vaccines

AC1 and AC3 are viral vector COVID-19 vaccine candidates composed of an AAVrh32.33 capsid and an AAV2 inverted terminal repeat (ITR)-flanked transgene-expressing distinct SARS-CoV-2 S antigens (Figure 1A). AC1 encodes a full-length membrane anchored S protein (Wuhan strain) mutated to abrogate the S1/S2 furin cleavage site and to lock its pre-fusion conformation to allow for optimal RBD antigenicity (Walls et al., 2020; Wrapp et al., 2020). AC3 expresses the secreted S1 subunit of the Wuhan S protein (Figure 1A). Expression of the S transgene was detected for each AAVCOVID candidate *in vitro* by transfection and transduction of both candidates (Figures S1A and S1B). AAVrh32.33 is a previously described rhesus derived AAV serotype. It is most closely related to AAV4 but phylogenetically divergent from the AAVs that are most commonly circulating and used as gene therapy vectors in humans (Figure 1B). Previously it was shown in an extensive human epidemiological study that the seroprevalence of antibodies to AAVrh32.33 is minimal (Calcedo et al., 2009). Consistent with these findings, 50 plasma samples collected from healthy human donors demonstrated highly reduced neutralizing antibody prevalence to AAVrh32.33 as compared with AAV8 and AAV2, with 6% of samples with titers of 1:20 or above compared with 22% and 28%, respectively (Figure 1C).

Given the need for scaled production of vaccines, we evaluated whether AC1 and AC3 manufacturing was feasible at scale. Multiple research-grade productions of AC1 were comparable, and slightly reduced for AC3, to the high-yielding AAV8 and AAV9 serotypes (Figure S1C). Final product showed consistent biophysical identity in the preparations (Figure S1D) (Pacouret et al., 2017). To evaluate the manufacturing performance at scale, a previously established scalable process developed by Novartis Gene Therapies was used to produce AC1 and AC3 (Figure 1D). Specifically, approximately 1.5 × 10^10^ HEK293 were seeded in a fixed-bed bioreactor (PALL iCellis 500), grown for 4 days, and then co-transfected with the AAVCOVID ITR plasmids, pKan2/rh32.33 for the AAV capsid and pALDX80 as helper. At harvest, yields for AC1 and AC3 were above 7 × 10^15^ genome-containing particles or genome copies (gc). Subsequent purification included multiple tangential flow filtration (TFF), an ion-exchange chromatography, and a cesium chloride density gradient step to eventually recover between 21% and 26% in the final drug substance. Of note, given the expedited nature of these studies, less than 2 weeks of process development studies were performed to successfully adapt the existing scalable production system to AAVrh32.33.

To interrogate the cold-chain requirements for storage and transportation of AAVCOVID in liquid formulation, toxicology grade AC1 product generated with the iCellis process was aliquoted (n = 5) and stored at different temperature conditions (−80°C, 4°C, or 25°C) for 4 and 12 weeks. Physical stability was assessed by ddPCR for genomic titer (Figure 1E) and differential scanning fluorimetry (Pacouret et al., 2017) for capsid integrity (Figures S1E and S1F). Functional stability was measured via RBD-IgG ELISA (Figure 1F) and pseudovirus neutralization (Figure 1G) on serum collected 3 weeks following single 10^11^ gc dose IM immunization of female C57BL/6. Storage at 25°C for 12 weeks led to reduction of genomic titer, capsid instability, and a loss of potency. AC1 was found stable in liquid formulation at all temperature conditions for 1 month, and for at least 12 weeks at −80°C and 4°C (Figures 1E–1G, S1E, and S1F).

### A single dose of AAVCOVID induces high and durable immune response in mice

**Figure 2:**
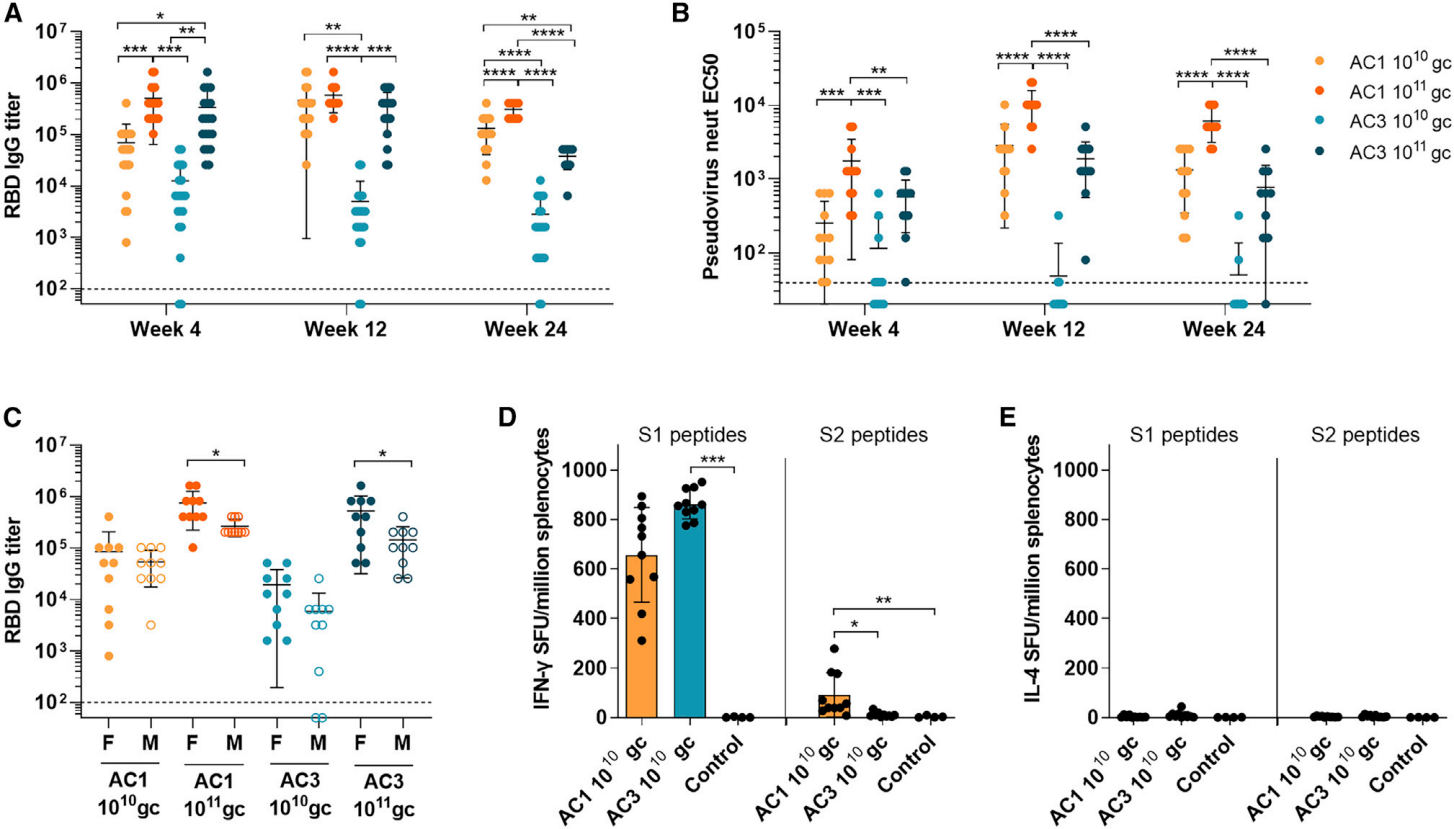
Quantitative assessment of humoral and cellular responses against SARS-CoV-2 spike in mice C57BL/6 mice (7–8 weeks old) were injected IM with two doses (10^10^ gc and 10^11^ gc) of AC1 or AC3(A) SARS-CoV-2 RBD-binding IgG titers, n = 20 (10 females and 10 males). (B) Pseudovirus neutralizing titers (EC_50_ international units (IU)/mL) (6 females and 6 males per group). (C) SARS-CoV-2 RBD-binding IgG titers in males and females 4 weeks after vaccination. (D and E) Spot-forming units (SFU) detected by IFN-γ (D) or IL-4 (E) ELISPOT in splenocytes extracted from animals 6 weeks after vaccination with 10^10^ gc of AC1 or AC3 and stimulated with peptides spanning SARS-CoV-2 spike protein for 48 h. The dotted lines indicate the lower detection limit of the assays. All data are represented as mean ± SD. (A and B) One-way ANOVA and Tukey’s post-test. (C) Student’s t test. (D and E) Kruskal Wallis and Dunn’s post-test. ^∗^p < 0.05, ^∗∗^p < 0.01, ^∗∗∗^p < 0.001, ^∗∗∗∗^p < 0.0001. See also Figures S2 and S3.

The immunogenicity of AC1 and AC3 following a single injection at a low and high dose of 10^10^ and 10^11^ gc, respectively, in the gastrocnemius muscle was evaluated in 6–7-week-old C57BL/6 (Figure 2) and BALB/C (Figures S2C–S2E) mice of both genders. SARS-CoV-2 RBD-binding IgG antibody levels were monitored by ELISA at regular intervals (Figures 2A and S2C), as were neutralizing antibody levels assayed using a SARS-CoV-2 spike pseudotyped lentivirus (pseudovirus) inhibition-of-transduction method (Figures 2B and S2D).

Dose-dependent potent binding and neutralizing responses were detected from a single-dose administration of AC1 or AC3 that persisted for 6 months in C57BL/6 (Figures 2A and 2B). Overall, AC1 at high doses induced a significantly higher level of RBD-binding and pseudovirus neutralizing antibody titers than AC3. Full seroconversion was achieved for both AC1 doses and the high AC3 dose but was partial for AC3 at the low dose (Figure 2A). Immunogenicity was lower by 2–3-fold in males versus female mice 4 weeks after vaccination for both candidates at high dose (Figure 2C), but no significant differences were found in later time points (data not shown). Neutralizing antibody kinetics lagged by approximately a week to stabilize after 4 weeks (Figures 2B and S2A). Across all candidates and time points, RBD-binding and neutralizing titers showed modest correlation and illustrated that AC1 achieved higher levels of neutralizing antibody compared with AC3 (Figure S2B). Studies in BALB/c confirmed the findings in C57BL/6 mice, however, overall had lower antibody titers. Distinct from C57BL/6 mice, at low does AC1 and AC3 led to similar levels of RBD-binding titers (Figures 2C–S2E). Antibody isotyping was analyzed to assess Th1/Th2 responses, which is an important measurement since dysregulated Th2 responses have previously been associated with vaccine enhanced disease (Lee et al., 2020; Tseng et al., 2012). This analysis suggested a balanced Th1 response stimulated by AC1, yet a more Th2 skewed AC3 response in BALB/c mice (Figure S3A).

Cellular responses were quantified by ELISPOT in splenocytes from low-dose AC1 and AC3 immunized C57BL/6 animals 6 weeks after vaccination. IFN-γ ELISPOT revealed a robust response against peptides spanning the S1 subunit (Figure 2D), while lower responses were detected against the S2 subunit only in the AC1 vaccinated group. Minimal IL-4 responses were seen by IL-4 ELISPOT (Figure 2E; ELISPOT positive control results are shown in Figures S3F and S3G). BALB/c animals treated with the high dose of AC1 and AC3 showed same cellular response pattern by ELISPOT 4 weeks after vaccination (Figures S3B–S3E).

### Durable immunogenicity in NHP from a single-dose injection

**Figure 3:**
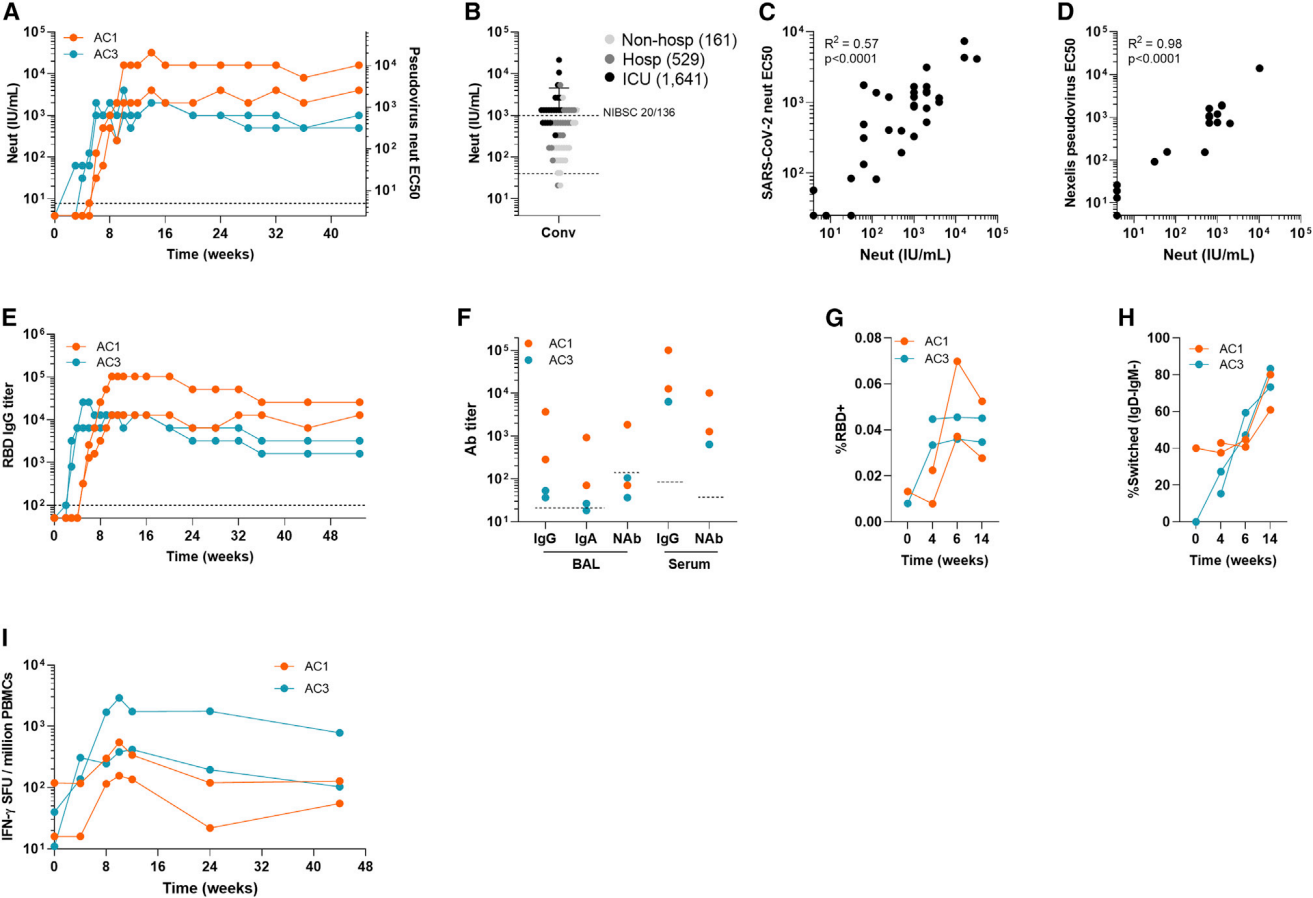
Characterization of humoral and cellular immune responses in rhesus macaque durability study Rhesus macaques (n = 2/group, 1 female and 1 male) were injected IM with 10^12^ gc of AC1 or AC3.(A) Pseudovirus neutralizing antibody titers (international units (IU)/mL in the right y axis and reciprocal serum dilution in the left y axis) 44-week follow-up. (B) For reference, pseudovirus neutralizing antibody titers (IU/mL) in 60 convalescent human plasma samples of patients with different disease severity. The Geometric mean titer (GMT) is shown in the figure legend for each cohort of convalescent plasma. The dotted line indicates the lower detection limit of the assay. (C and D) Correlation between pseudovirus neutralization (IU/mL) and live SARS-CoV-2 neutralization titers (C) and VSV pseudovirus neutralization titers measured at the reference laboratory Nexelis (Laval, Canada) (D). Pearson’s correlation coefficient was calculated to assess correlation. (E) SARS-CoV-2 RBD-binding IgG titers 53-week follow-up. (F) RBD-binding IgG and IgA and pseudovirus neutralizing titers in bronchoalveolar lavage (BAL) samples harvested on week 20 after vaccination, in comparison with IgG and neutralizing titers detected in serum at the same time point. (G) Frequency of RBD-binding B cells with a memory phenotype (CD27^+^ or CD27^−^IgD^−^) in the peripheral blood B cell compartment as measured by flow cytometry. (H) Frequency of isotype-switched (IgD^−^IgM^−^) phenotype within RBD-binding memory B cells as measured by flow cytometry. (I) Quantification of IFN-γ SFUs by ELISPOT in peripheral blood mononuclear cells (PBMC) samples collected at different time points and stimulated with peptides specific for each transgene. The dotted lines indicate the lower detection limit of the assay. See also Figure S4.

To qualitatively assess the immunogenicity of AAVCOVID in humans, rhesus macaques (n = 2 per vaccine candidate) were IM injected with 10^12^ gc of AC1 and AC3 and monitored for now up to 1 year in an ongoing study. Animals tolerated the vaccine dose well, with no temperature elevations or local reactogenicity based on clinical examinations, complete blood counts and chemistry, or cytokine analysis (data not shown). Regular phlebotomies were performed to assess RBD-binding, pseudovirus neutralizing, and live SARS-CoV-2 neutralizing antibody titers in serum and bronchoalveolar lavage (BAL) (Figures 3A–3F) and B and T cell analyses from PBMCs (Figures 3G–3I).

AC1 and AC3 induced high SARS-CoV-2 RBD-binding and neutralizing antibody responses from a single-dose injection (Figures 3A and 3E). Antibody kinetics differed for both candidates, similar to the mouse; AC3 reached near-peak level binding titers at week 4 and neutralizing antibodies leveled off 1 or 2 weeks later. On the other hand, binding antibodies were detected for AC1 at week 5, increased until it peaked at week 11. Similar to AC3, kinetics for neutralizing titers trailed the binding ones by 1 or 2 weeks. Importantly, neutralizing titer levels remained at peak for at least 11 months (1:1,280 and 1:10,240 [or 2,000 and 16,000 IU/mL]) and in range of convalescent titers (3,016 ± 4,767 IU/mL) from patients that underwent ICU admission (Figure 3B). Both AC1 animals showed higher neutralizing antibody levels than those immunized with AC3 (Figure 3A). Neutralization was further validated and benchmarked by live SARS-CoV-2 plaque neutralization (Figure 3C) and VSV pseudovirus at the Nexelis BMFG and CEPI reference laboratory (Laval, Canada) (Figure 3D). A BAL was performed at 5 months following immunization and showed detectable levels of RBD-binding IgG, IgA, and neutralizing antibodies for AC1, but not for AC3 (Figure 3F).

Flow cytometry analysis of the frequency of RBD-binding memory B cells (MBCs) demonstrated an increase up to week 6 following vaccination in all animals that was sustained at least through week 14 (Figures 3G, 3H, and S4A). Surface immunoglobulin isotype analyses found an early bias toward generation of IgM-expressing MBCs, whereas isotype-switched (IgD^−^IgM^−^) MBCs dominated the SARS-CoV-2 RBD-specific response by week 14 (Figure 3H). These findings suggest durable induction of RBD-specific MBCs by both AC1 and AC3.

T cell responses to SARS-CoV-2 spike peptide pools were analyzed by IFN-γ ELISPOT (Figure 3I), and responses were detectable starting on week 4, peaked around week 10, and were still detectable on week 44 (month 11) post-vaccination. Higher response was measured in one of the AC3 animals, which also showed robust memory T cell profile by intracellular staining of PBMCs (Figures S4B–S4E).

Given the low N number in this study, these results illustrate the qualitative potential of AC1 and AC3. Based on the superior immunogenicity in mice and (in a limited manner in) macaque, its modestly higher manufacturing yields, and its ability to induce a proportionally more neutralizing response, AC1 was selected as a lead candidate to progress in additional well-powered studies.

### AC1 provides protection in a cynomolgus macaque SARS-CoV-2 challenge model

**Figure 4:**
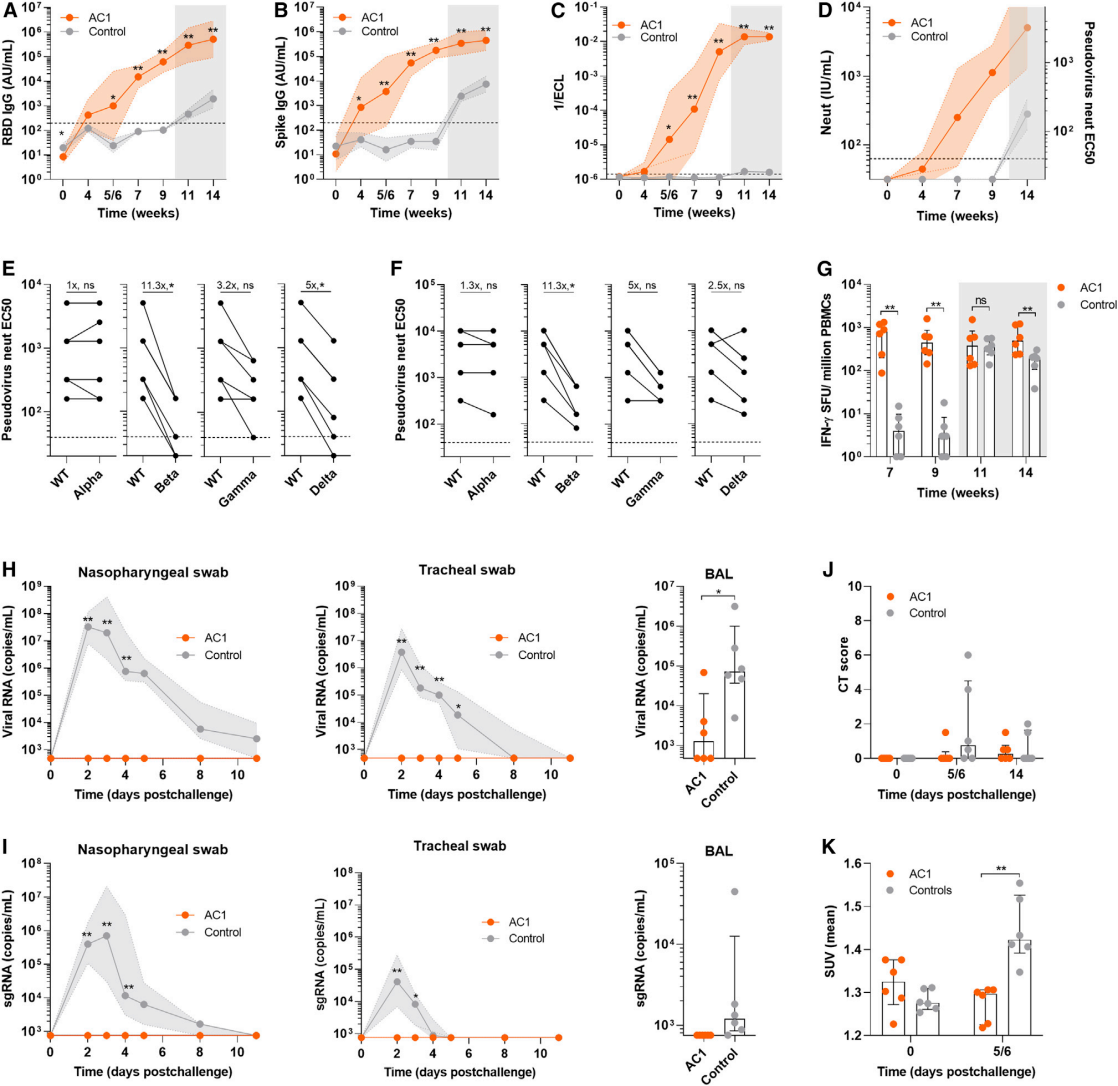
Protection from SARS-CoV-2 challenge in cynomolgus macaques vaccinated with AC1 Cynomolgus macaques (n = 6) were injected with 10^12^ gc of AC1 or sham (Control). After 9.5 weeks all animals received an intranasal (10%) and intratracheal (90%) inoculation with 10^5^ pfu of SARS-CoV-2 (BetaCoV/France/IDF/0372/2020). (A and B) SARS-CoV-2 RBD and spike ectodomain-binding IgG (absorbance units [AU]/mL) (C) Measurement of antibodies that inhibit binding of spike to ACE2 in an *in vitro* binding inhibition assay. (D) Pseudovirus neutralizing antibody titers (IU/mL in the right y axis and reciprocal serum dilution in the left y axis). (E and F) Neutralization of SARS-CoV-2 Alpha, Beta, Gamma, and Delta variants of concern (VOC) on 9 weeks post-vaccination (E) and on week 14 (week 4 post-challenge) (F) in a pseudovirus neutralization assay. Fold-change of geometric mean titers is displayed. Wilcoxon matched-pairs signed rank test was used to compare titers with the WT and VOC. (G) Quantification of IFN-γ SFUs by ELISPOT in PBMC samples stimulated with peptides of SARS-CoV-2 spike protein. (H) SARS-CoV-2 viral RNA copies in nasopharyngeal (left) and tracheal swab (middle) at several time points after 10^5^ pfu SARS-CoV-2 challenge and in bronchoalveolar lavage (BAL) fluid (right) at day 3 after challenge. (I) SARS-CoV-2 subgenomic RNA quantification (copies/mL) in copies fluids described in (H). (J) CT score in lungs of control and vaccinated animals before and after challenge. Scores were calculated based on lesion type (scored from 0 to 3) and lesion volume (scored from 0 to 4) for each lobe. (K) Measurement of lung lymph node (LN) activation measured by PET as mean standardized uptake values (SUV mean) before and after challenge. The dotted lines indicate the lower detection limit of the assay. Gray-shaded areas correspond to post-challenge time points. (A–D) Data are represented as geometric mean titer (GMT) ± geometric SD. Mann-Whitney test was used to compare vaccinated and control groups. (G–K) Data are represented as median ± interquartile range. Mann-Whitney test was used to compare vaccinated and control groups. ^∗^p < 0.05, ^∗∗^p < 0.01. See also Figures S5 and S6.

AC1 was subjected to a SARS-CoV-2 viral challenge study in cynomolgus macaque. Two groups of animals (n = 6 per group) were injected IM with either 10^12^ gc of AC1 or vehicle (control). After 9.5 weeks, both groups were inoculated via combined intranasal (10%) and intratracheal (90%) route with 10^5^ pfu of SARS-CoV-2 (BetaCoV/France/IDF/0372/2020) (Lescure et al., 2020). This isolate presented a mutation in spike (V367F) shown to increase infectivity (Ou et al., 2021) and sensitivity to neutralizing antibodies (Li et al., 2020). Humoral and cellular immunogenicity was monitored throughout the study (Figures 4A–4G). Quantification of viral and subgenomic SARS-CoV-2 RNA in upper and lower respiratory tract was performed by qPCR (Figures 4H and 4I). Lesions in the lung and inflammation were monitored by computed tomography (CT) and positron emission tomography (PET) and scored to assess the protection conferred by AC1 (Figures 4J, 4K, and S6).

RBD- and Spike-binding antibodies became detectable after week 4 following single-dose vaccine injection and seroconversion was complete at week 7 yet continued to rise at the challenge at week 9 (Figures 4A and 4B). ACE2-Spike binding inhibition titers followed a similar kinetics, indicative of the presence of neutralizing antibodies which was confirmed using a cell-based pseudovirus assays (Figures 4C and 4D). Cross-reactivity of antibodies in the sera harvested on week 9 (pre-challenge, Figure 4E) and week 14 (4 weeks post-challenge, Figure 4F) to 4 SARS-CoV-2 variants of concern (Alpha [B.1.17] emerged in the UK, Beta [B.1.351] in South Africa, Gamma [P.1] in Brazil, and Delta [B.1.617.2] in India) was tested in a pseudovirus neutralization assay. Sera from AC1-treated animals neutralized Alpha variant at same level as the parental SARS-CoV-2 spike pseudovirus. However, neutralization was reduced by 11.3-fold against Beta, 3–5-fold against Gamma, and 2–5-fold against Delta, similar in magnitude to the reduction observed in plasma from convalescent patients that were infected with wild-type SARS-CoV-2 (Figure S5K). Importantly, all animals showed some degree of neutralization of all VOCs on week 14 (Figure 4F). IFN-γ ELISPOT showed robust cellular response in PBMCs in all AC1 animals by week 7, which were maintained at week 9 (Figure 4G).

In the unvaccinated control animals, the challenge led to high viral loads in the upper respiratory tract and in the lung (Figures 4H, S5A, and S5B). Active viral replication was detected in all control animals in the upper and lower tract by quantification of subgenomic RNA (sgRNA) (Figures 4I, S5C, and S5D). In contrast to the AC1 immunized group, no detectable viral RNA was detected in the upper respiratory tract animals (Figure 4H), except for low but detectable breakthrough in 2 animals for viral RNA, and for sgRNA in one of them (Figures S5A–S5D). In BAL, 3 animals had detectable levels of viral RNA (Figure 4H), but levels of RNA were still significantly lower when compared with controls. No sgRNA was detected in BAL of vaccinated animals (Figure 4I). These data indicate that the protection conferred by AC1 is profound from viral replication in both upper and lower respiratory tract.

Lung damage was assessed by CT scan analysis (Figure 4J), and no major lesions were observed in the immunized animals. In contrast, control animals presented larger and more severe lesions in 2 animals. Lung draining lymph node activation was analyzed by [^18^F]-FDG uptake using PET scan, and all control animals presented an increased activation after challenge, indicative of SARS-CoV-2 infection (Figure 4K). Examples of images obtained by PET scan after challenge from non-vaccinated NHPs are presented (Figure S6). Animals that received AC1, however, showed no change in inflammatory responses upon challenge, most likely due to complete neutralization of the virus and suggestive of sterilizing protection (Figure 4K). Additionally, control animals showed a decrease in circulating lymphocyte count on day 2 following viral challenge, which is also observed in COVID-19 patients (Zhu et al., 2020c), while immunized animals did not have circulating lymphocyte levels drop, another indicative of protection to SARS-CoV-2 infection (Figure S5E). Finally, control animals developed antibody and T cell responses to SARS-CoV-2 after the challenge, expected following an active infection (Figures 4A–4C, 4G, S5F, and S5J). However, AC1 immunized animals did not show overall increase in these parameters, except in RBD-binding antibodies 4 weeks post-challenge (Figures 4A–4C, 4G, S5F, and S5J).

### NHPs develop limited neutralizing antibody response to AAVrh32.33 capsid that is not cross-reactive with clinically used AAV serotypes

Viral vector vaccines are known to induce antibody responses to vector components, here, the AAV capsid, which can block the efficacy of subsequent administrations of the same or similar viral vaccine or gene therapy (Greig et al., 2016; Majowicz et al., 2017). Samples from the rhesus immunogenicity study above were analyzed for *in vitro* neutralization of AAVrh32.33, used in AC1, and serotypes AAV1, 2, 5, 8, and 9, which are commonly used in gene therapy studies. Table 1 illustrates AAVrh32.33 neutralizing antibodies developed with slow kinetics and to relatively low levels. Importantly, no cross-reactive neutralizing antibody responses were detected to AAV1, 2, 5, 8, or 9. Furthermore, compared with baseline sample, no significant AAV capsid-specific cellular response was detected in PBMCs collected from animals 4 and 8 weeks after IM AC1 or AC3 injection (Figure S4F).

**Table 1:** Neutralizing AAV responses elicited by AAVCOVID in NHP

Animal	Time (weeks)	AAVrh32.33	AAV1	AAV2	AAV5	AAV8	AAV9
**AC1 NHP#1**	**0**	< 5	< 5	< 5	< 5	5	< 5
	**4**	< 5	< 5	< 5	< 5	< 5	< 5
	**8**	< 5	< 5	< 5	< 5	< 5	< 5
	**12**	20	< 5	< 5	< 5	< 5	< 5
	**14/16/20**	80/80/160	N/A	N/A	N/A	N/A	N/A
**AC1 NHP#2**	**0**	< 5	< 5	< 5	< 5	< 5	< 5
	**4**	10	< 5	< 5	< 5	< 5	< 5
	**8**	160	< 5	< 5	< 5	< 5	< 5
	**12**	320	< 5	< 5	< 5	< 5	< 5
	**14/16/20**	320/320/320	N/A	N/A	N/A	N/A	N/A
**AC3 NHP#3**	**0**	< 5	< 5	< 5	< 5	< 5	< 5
	**4**	< 5	< 5	< 5	< 5	< 5	< 5
	**8**	40	< 5	< 5	< 5	< 5	N/A
	**12**	160	< 5	< 5	< 5	< 5	< 5
	**14/16/20**	160/160/160	N/A	N/A	N/A	N/A	N/A
**AC3 NHP#4**	**0**	< 5	< 5	< 5	< 5	< 5	< 5
	**4**	5	< 5	< 5	< 5	< 5	< 5
	**8**	40	< 5	< 5	< 5	< 5	< 5
	**12**	80	< 5	< 5	< 5	< 5	< 5
	**14/16/20**	320/320/160	N/A	N/A	N/A	N/A	N/A

Neutralizing antibody titers against the injected vector (AAVrh32.33) and cross-reactive neutralizing against other serotypes (AAV1, AAV2, AAV5, AAV8, and AAV9) monthly after vaccination of rhesus macaques.

### AAVCOVID genome persistence and expression is self-limiting and focal to the injection site

**Figure 5:**
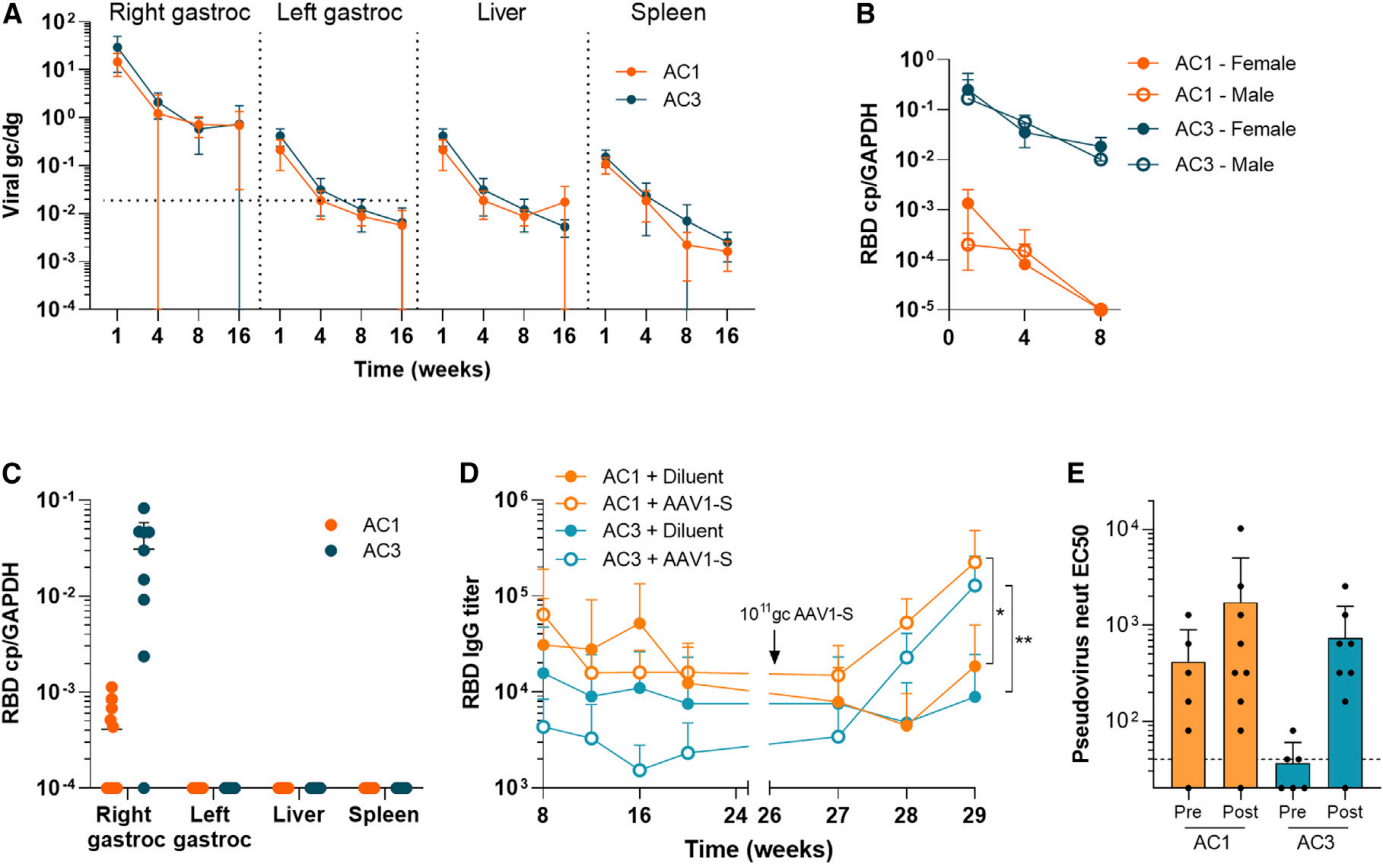
Pharmacology of AAVCOVID vaccines in mice (A) Quantification of vector genome copies (DNA, genome copies/diploid genome or gc/dg) in the right gastrocnemius (right gastroc) or injection site, left gastrocnemius (left gastroc) or contralateral muscle, liver, and spleen on weeks 1, 4, 8, and 16 after the administration of 10^11^ gc of AC1 or AC3 in C57BL/6 animals (n ≥ 6, 3–5/gender). Horizontal dotted lines indicate background levels for right and left gastrocnemius muscles. Liver and spleen had not detectable background. (B) Quantification of transgene expression (RBD copies/GAPD copies or RBD cp/GAPDH) in the right gastrocnemius muscle. (C) Quantification of expression (RBD copies/GAPD copies or RBD cp/GAPDH) in the injection site (right gastroc), contralateral muscle (left gastroc), liver and spleen on week 16. (D and E) SARS-CoV-2 RBD-binding antibody titer 29-week follow-up in BALB/c mice (n ≥ 9) vaccinated with 10^10^ gc of AC1 and AC3 on week 0 and revaccinated with 10^11^gc of AAV1-S (open circles) or diluent (solid circles) on week 26. (E) Pseudovirus neutralizing titers in animals described in (D) before and after the boost with AAV1-S. (A–E) Data are represented as mean ± SD. (D and E) Student’s t test. ^∗^p < 0.05, ^∗∗^p < 0.01.

AAVCOVID transgene-specific DNA and mRNA analysis of various C57BL/6 tissues of animals that received a single intramuscular injection was performed to establish biodistribution, the persistence of vector genomes, and the kinetics of transgene expression (Figure 5). Previously, an AAVrh32.33 expressing a non-self-transgene, when injected intramuscularly in mice, showed declining transgene expression over time that was associated with increasing inflammatory infiltrates at the injection site several weeks after injection (Mays et al., 2009, 2013). This is in stark contrast to other AAVs expressing the same transgene, which led to stable transgene expression and minimal local inflammation (Mays et al., 2009, 2013). In this experiment, C57BL/6 mice were injected with 10^11^ gc in the right gastrocnemius muscle. Animals were euthanized 1, 4, 8, and 16 weeks after vaccination and tissues were analyzed for vector genome copies and transgene expression. As observed in Figure 5A, vector genome copies in the injected muscle decreased more than 20-fold from week 1 to week 16. AC3 transgene expression declined similarly. Remarkably, AC1 transgene expression was close to background levels and over 10-fold lower than AC3 expression, possibly due to the different promoter in both candidates (Figure 5B). Gene transfer and transduction levels of the contralateral gastrocnemius muscle, liver, and spleen demonstrated 10- to 100-fold less vector DNA at week 1 than measured in the injected muscle with a steady decline of vector DNA and RNA, to undetectable RNA levels by week 16 (Figure 5C).

In order to address the potential concern that prolonged (yet waning) exposure to antigen may lead to a desensitizing response, a prime-boost study was performed with 10^10^gc of AC1 and AC3 as prime. 26 weeks after prime immunization with AC1, animals were boosted with AAV1-S, an AAV1 capsid to avoid neutralizing anti-AAVrh32.33 antibody interference that encodes a full-length SARS-CoV-2 S (AAV1-S). In comparison with control groups that did not receive a boost, RBD-binding and neutralizing antibody responses were functionally recalled after AAV1-S administration (Figures 5D and 5E).

## Discussion

Early in 2020, global health and economic vitality was disrupted by the pandemic of COVID-19. A multitude of vaccine technologies have been pursued following the identification of SARS-CoV-2 as the etiological agent (Zhou et al., 2020). In a remarkable effort within 12 months, several vaccines were found to be safe and effective and have been approved by health regulators in various territories. In the early phase of the rollout, vaccine distribution has been highly uneven across the globe primarily due to economic factors and limiting manufacturing supply. Large-scale vaccine campaigns have successfully been mounted in the West; however, these are not without initial, and in some cases remaining, challenges. Once substantial vaccine doses are available for low- and middle-income countries, the logistical needs of current vaccines are expected to complicate and prolong the buildup of population immunity at the needed pace; most vaccines currently available require 2 doses and require some type of cold storage. The need for more and more deployable vaccine options thus remains exceptionally high. Ideally, these options should utilize manufacturing streams distinct from current vaccines, be effective from a single dose, and have minimal to no cold-chain requirement. This need is further amplified due to the uncertainty around emerging SARS-CoV-2 variants and vaccine protection, the durability of protection from current vaccines, and vaccine hesitancy.

Here, we describe the AAV-based vaccine candidate AC1 that seeks to address a number of these biological and logistical limitations. AC1, a SARS-CoV-2 Spike antigen and gene-based viral vector vaccine was found protective in non-human primates from a single low-dose intramuscular injection (Figure 4). Immunogenicity of AC1 and AC3 in mouse and non-human primate models further demonstrates potent and durable (up to 11 month) neutralizing antibody responses complemented by strong T cell immunity from a single-dose administration. These characteristics are shared with several other gene-based vaccine approaches presented to date, likely due to their ability to express the antigen endogenously and thus allow for direct antigen presentation. Additionally, AC1 manufacturing leverages an established process at scale currently used in the AAV gene therapy industry (Figure 1). Importantly, due to the hardy nature of the AAV capsid, it remains stable and active when stored at room temperature for a month (Figure 1).

AC1 provides the immunogenicity and protection from challenge in non-human primates from a single and low dose, whereas most SARS-CoV-2-approved vaccines, except for Ad26.COV2.S, require 2 injections for full efficacy (Corbett et al., 2020; Guebre-Xabier et al., 2020; Mercado et al., 2020; van Doremalen et al., 2020b; Vogel et al., 2021). In the upper airway of immunized cynomolgus macaques, minimal to no virus was detected, and replication was nearly fully suppressed, suggesting that AC1 may also reduce viral transmission (Figure 4). Protection in the airway is not easily achieved, including in viral challenge experiments from several currently approved vaccines (Corbett et al., 2020; van Doremalen et al., 2020b; Vogel et al., 2021). Previously, Ad26 with a highly analogous antigen design as in AC1, was shown to similarly provide protection from a single shot (Mercado et al., 2020), in contrast most other gene-based modalities, e.g., mRNA vaccines, require a second dose to achieve comparable levels of immunogenicity (Corbett et al., 2020; Guebre-Xabier et al., 2020; van Doremalen et al., 2020b; Vogel et al., 2021). Remarkably, clinical data from the Pfizer/BioNTech and Moderna vaccines illustrate a protection benefit from the first dose; however, no data are available on the durability of that protection (Baden et al., 2021; Moghadas et al., 2021; Polack et al., 2020), and the data are limited in providing cross-protection to emerging VOCs (Planas et al., 2021).

While a broadly accepted correlate of protection has yet to be established, the level of neutralizing antibody responses in serum is seen as a major component of the immunity required for protection (Jin et al., 2021; McMahan et al., 2021). It remains, however, difficult to compare the various readouts of immunogenicity between vaccines, laboratories, and assays. We went to significant length to reference our neutralizing antibody readout and benchmark the levels from AC1 and AC3 to allow for these comparisons. The levels from AC1 are in the upper ranges of convalescence, similar to the levels of patients that survived an ICU stay. AC1, when compared with data in non-human primates from other vaccines that later demonstrated protection in humans, provides similar levels of neutralizing antibodies to SARS-CoV-2. Furthermore, using international standards and reference laboratories, AC1 data can directly be compared across comparable studies with the vaccine data of other groups.

To prevent the need or limit the number of boost administrations, durable protection from a vaccine is desirable. AC1, in mice and non-human primates, demonstrates at least for 6 months in mice and 11 months in NHP neutralization to be stable at peak levels. The available datasets on durability for other vaccines are minimal in animal models and slowly emerging from clinical studies and their use in the population as approved vaccines. Data from the Moderna mRNA vaccine show antibody levels to remain high for up to 4 months, however on a gradual decline from peak levels at 2–4 weeks after the second dose (Widge et al., 2021).

What drives the single-dose potency and durability of AAVCOVID remains unclear. We hypothesize antigen expression from AAVCOVID is more long lived than other gene-based vaccine modalities, allowing for germinal centers to mature and amplify B cells (Gaebler et al., 2021). Unlike mRNA, which has a short half-life, viral vector vaccines such as adenoviral and AAV-based vaccines may share the single-dose benefit by the longer antigen expression they support. Our data support this hypothesis: vector genome copies and expression peaks 7 days following intramuscular injection from when it starts waning over a period of several weeks to be reduced over 10-fold. By week 8, AC1 expression is close to undetectable although a low level of vector genomes remains present at the injection site. In tissues outside the injection site, levels are negligible at week 8 and undetectable at week 16, illustrating the limited biodistribution of AAVrh32.33 following IM injection. Extended maturation of B cells in germinal centers due to more persistent antigen may have other benefits, as it has been described to lead to expanded antibody repertoire, higher antibody affinity, and a broader array of epitope recognition, e.g., for broader neutralization of variants of concern (Muecksch et al., 2021). In a vaccine setting, previously Cirelli et al. demonstrated the benefits of slow delivery immunization in the context of HIV; as opposed to multiple injections or an osmotic pump, AAV may provide a more facile delivery to achieve a similar outcome (Cirelli et al., 2019). Extended antigen exposure however may lead to concerns of desensitizing responses that are clearly undesirable in a vaccine context. Previously, we demonstrated that in the context of HIV in non-human primates, the AAVrh32.33 vaccine vector technology led to functional recall in an adenoviral boost of both antibody and T cell repertoire. Here, we show AC1-induced antibody levels can be boosted by a second immunization with a different AAV vector expressing the same spike antigen (Figure 5D).

In addition to a single-dose benefit, AC1 may further facilitate vaccine deployment and delivery. AAV is a known thermostable viral vector and, in the context of AC1, demonstrates physical and biological stability following 1 month of storage at 4°C and 25°C. In addition, the methods for manufacturing and analysis used here for AC1 (and AC3) largely mirror an established, validated, and previously scaled process of Novartis Gene Therapies. As AAV is a clinical and commercial-stage technology, the extensive experience and capabilities within the industry may allow for accelerated development compared with other vaccine modalities. While AAV gene therapy drugs on the market are known to be costly, an AAV vaccine cost is expected to be competitive given the expected low-dose requirement and alternate pricing paradigms for vaccines versus rare disease drugs. An additional consideration for the distribution of viral vector vaccines is the pre-existing immunity to the vector capsid. Unlike most AAVs and adenoviruses that are endemic in humans, the AAVrh32.33 technology used for AC1 and AC3 has no relevant levels of anti-AAV neutralization (Figure 1 and Calcedo et al., 2009), suggesting that broad protection across humans can be achieved without interference of pre-existing immunity to the vector.

AAV has been extensively studied for 40 years, including clinically for over 25 years. Its applications to data are primarily in the field of therapeutic gene transfer and have now led to 3 approved AAV-based gene therapy products (Bennett et al., 2016; Mendell et al., 2017; Stroes et al., 2008). Except for one clinical study exploring the use of AAV2 expressing HIV-1 gag, protease, and part of the reverse transcriptase proteins as an AIDS vaccine (Mehendale et al., 2010; Vardas et al., 2010), the majority of AAV studies for preventative immunization are laboratory based (Li et al., 2012; Lin et al., 2009; Xin et al., 2006; Zhu et al., 2019). Collectively, the safety profile of AAV was highly favorable, even in applications of AAV that rely on systemic administration at doses of 10^14^ per human dosing (or approximately 2 × 10^12^ gc/kg) (High and Roncarolo, 2019). Dose-related safety findings have been made at doses exceeding this level, particularly when vector was delivered systemically (Hinderer et al., 2018). Importantly, for use for immunization of a healthy population, the risk versus benefit for a vaccine is substantially different compared with the use of AAV in a gene therapy setting. Further studies are needed to gauge the safety of AC1 at the established human doses, as a prerequisite to human testing.

By design, AC1 was constructed as a gene-based vaccine by incorporating an antigenic transgene into the choice of the specific AAVrh32.33-vaccine-appropriate AAV technology. Here, we were able to confirm these cornerstone attributes on AC1 and AC3, including the seroprevalence (Figure 1C), clearance kinetics (Figures 5A and 5B), and immunogenicity in mouse and non-human primate (Figures 2, 3 and 4). Elegantly, given the divergent structural and serological profile of the AAVrh32.33, no cross-reactivity to common vectors used in gene therapy was observed in non-human primate studies, suggesting that AC1 immunization would not interfere with possible future AAV gene therapy applications (Table 1; Figure S6). AC1 and AC3 however do induce neutralizing antibodies to the AAVrh32.33 capsid, possibly interfering with subsequent AAVCOVID repeat dosing (e.g., boost or variant vaccine). Previously, levels up to 1:320 of AAV8 allowed for an AAV8 re-administration to be performed without inhibition (Greig et al., 2016). However, these would have to be repeated in the context of AAVrh32.33 and AC1. If repeat administration is needed, one could seek a heterologous prime-boost approach with other viral vector vaccines (e.g., adenoviral vectors) that suffer from a similar limitation. The AAVrh32.33 technology here demonstrates its utility as a preventative vaccine for COVID19, and its unique attributes may be applicable to other pathogens or immunization targets.

The World Health Organization has developed early in the pandemic a preferred target product profile for a COVID-19 vaccine. These include a single-dose primary series followed by lower frequency booster doses, as well as stability at higher storage temperatures, as these are thought to “greatly enhance vaccine distribution and availability” (https://www.who.int/publications/m/item/who-target-product-profiles-for-covid-19-vaccines). Our studies on AC1 in animal models support its potential to protect from COVID-19 disease and infection from a single-dose immunization. In addition, based on the biology of AAV-based vaccines, the data suggest that AAV-based vaccines may lead to highly durable immunogenicity, possibly reducing the frequency and/or need for repeat dosing. Lastly, given the stability of the AAV capsid and its ssDNA genome, AC1 has been shown to be stable at ambient temperature for storage for several weeks. To date, the combination of these key attributes, as specified by the WHO, have not been met by any of the currently approved vaccines. Without a clear resolution of the current epidemic at a global scale, a continued development of second-generation vaccine candidates such as AC1 is needed.

### Limitations of the study

Further and ongoing studies are needed to address limitations of the current work. These studies describe the characteristics of AAVCOVID in animal models and thus illustrate the potential for human use. To establish human safety and efficacy, more extensive animal and human studies are needed. To establish the durability of the immunogenicity following a single-dose AAVCOVID immunization in a more conclusive way, better powered studies are needed. Lastly, while protection is established in our non-human primate studies at 10^12^ gc, dose-finding studies are required to further gauge safety, efficacy, gender differences, cost, and scalability aspects of this vaccine technology.

## STAR★Methods

### Key resources table

**Table:** 

REAGENT or RESOURCE	SOURCE	IDENTIFIER
**Antibodies**		

Peroxidase AffiniPure Rabbit Anti-Mouse IgG	Jackson ImmunoResearch	Cat# 315-035-045; RRID: AB_2340066
Rabbit Anti-Monkey IgG (whole molecule)-Peroxidase antibody	Sigma-Aldrich	Cat# A2054; RRID:AB_257967
SBA Clonotyping System-HRP kit	SouthernBiotech	Cat#5300-05; RRID:AB_2796080
anti-SARS-CoV-2 Spike monoclonal neutralizing antibody	GenScript	Cat#A02055; RRID: N/A

**Bacterial and virus strains**		

SARS-CoV-2 virus (hCoV-19/France/ lDF0372/2020 strain)	Lescure et al., 2020	EPI_ISL_410720 (GISAID ID)
SARS-CoV-2 (isolate USA-WA1/2020)	BEI resources	Cat#NR-52281
AAVCOVID19-1 (AC1)	Mass Eye and Ear Gene Therapy Vector Core	GenBank: MW408785
AAVCOVID19-3 (AC3)	Mass Eye and Ear Gene Therapy Vector Core	GenBank MW408786
AAVCOVID19-1 (AC1)	Novartis Gene Therapies	GenBank: MW408785

**Biological samples**		

Plasma from COVID-19 convalescent patients	Massachusetts General Hospital	N/A
WHO International Standard for anti-SARS-CoV-2	NIBSC	NIBSC 20/136

**Chemicals, peptides, and recombinant proteins**		

SARS-CoV-2 Wuhan RBD recombinant protein	Schmidt lab, Ragon Institute	N/A
SARS-CoV-2 Spike Glycoprotein D614G peptide pools	GenScript	Cat#RP30025
AC1 and AC3 transgene peptide pools	Mimotopes	N/A
Polyethylenimine or PEI	Polysciences	Cat#24765-2
Benzonase	EMD Millipore	Cat#1016970010

**Critical commercial assays**		

V-PLEX SARS-CoV-2 Panel 2 Kit	Mesoscale Discovery	Cat#K15383U and K12386U
Monkey IFNg ELISpot PRO kit	Mabtech	Cat#3421M-2APT

**Experimental models: Cell lines**		

HEK293 cell line	ATCC	CRL-1573
HEK293T cell line	ATCC	CRL-3216
HEK293T-ACE2 cell line	Hacohen lab	N/A

**Experimental models: Organisms/strains**		

BALB/c mouse strain (*Mus musculus*)	Jackson Laboratory	Stock#000651
C57BL/6J mouse strain (*Mus musculus*)	Jackson Laboratory	Stock#000664
Rhesus macaques (*Macaca mulatta*)	Sichuan Hengshu Bio-Tech Co., Ltd.	N/A
Cynomolgus macaques (*Macaca fascicularis*)	Cynologics Ltd	N/A

**Oligonucleotides**		

RBD Fw primer:GTGCAGCCAACCGAG	This paper	N/A
RBD Rv primer:ACACCTCGCCAAATGG	This paper	N/A
RBD TaqMan probe:6FAM- TCTATCGTG CGCTTTC-MGBNFQ	This paper	N/A
sgLeadSARSCoV2-F CGATCTCTTGTAG ATCTGTTCTC	Corman et al., 2020; Wolfel et al., 2020	N/A
E-Sarbeco-R primer ATATTGCAGCAG TACGCACACA	Corman et al., 2020; Wolfel et al., 2020	N/A
E-Sarbeco probe HEX-ACACTAGCCA TCCTTACTGCGCTTCG-BHQ1	Corman et al., 2020; Wolfel et al., 2020	N/A

**Recombinant DNA**		

pAAVCOVID19-1 (pAC1)	GenBank	MW408785
pAAVCOVID19-3 (pAC3)	GenBank	MW408786
pKan2/rh32.33	Vandenberghe lab	N/A
pALD-X80	Aldevron	N/A
psPAX2	Hacohen lab	N/A
pCMV-SARS2-Spike	Hacohen lab	N/A
pCMV-Lenti-Luc	Hacohen lab	N/A

**Software and algorithms**		

GraphPad Prism 8	GraphPad	https://www.graphpad.com/

**Other**		

Seracare SureBlue Reserve TMB Microwell Peroxidase Substrate solution	SeraCare	Cat#53-00-03
Seracare KPL TMB Stop Solution	SeraCare	Cat#50-85-06
Reporter Lysis Buffer	Promega	Cat# E4030
Pierce™ Firefly Signal Enhancer	Thermo Fisher Scientific	Cat#16180
D-luciferin	PerkinElmer	Cat#122799

### Resource availability

#### Lead contact

Further information and requests for resources and reagents should be directed to and will befulfilled by the lead contact, Luk H Vandenberghe (luk_vandenberghe@meei.harvard.edu).

#### Materials availability

Plasmid sequences generated in this study have been deposited to Genbank. AAVCOVID19-1 (AC1): Genbank MW408785 and AAVCOVID19-3 (AC3) GenBank MW408786.

### Experimental model and subject details

#### Mouse studies

All the mouse studies were performed in compliance with the Schepens Eye Research Institute IACUC. C57BL/6 (n=20, 10 males and 10 females) and BALB/c (n=20, 10 males and 10 females) were purchased from Jackson Laboratories. Animals were housed in standard BSL1 facilities, with 12-hour light cycles and free access to regular chow diet and water. 6-9 week-old mice were intramuscularly (right gastrocnemius muscle) treated at 10^10^ gc/mouse or 10^11^ gc/mouse. Serum samples were obtained by submandibular bleeds for humoral immune response analyses. At necropsy, several tissues were collected for analysis of vector presence and transgene expression.

#### NHP studies

Animal procedures performed in the rhesus macaques were approved by the Institutional Animal Care and Use Committee of the Children’s Hospital of Philadelphia. Rhesus macaques (*Macaca mulatta*) that screened negative for viral pathogens including SIV (simian immunodeficiency virus), STLV (simian-T- lymphotrophic virus), SRV (simian retrovirus), and B virus (macacine herpesvirus 1) were enrolled on the study. Animals were housed in an AAALAC International-accredited nonhuman primate research in stainless-steel squeeze back cages, on a 12-hour timed light/dark cycle, at temperatures ranging from 64-79°F (18-26°C). Animals received varied enrichment such as food treats, visual and auditory stimuli, manipulatives, and social interactions throughout the study. Four 3 to 7 year-old Rhesus macaques (*Macaca mulatta*) were treated with the clinical candidates (n=2/vector, 1 female and 1 male) intramuscularly at a dose of 10^12^ gc/animal. Serum and PBMC samples were obtained in regular intervals for several analyses of immunogenicity against SARS-CoV-2 Spike and AAVrh32.33.

Cynomolgus macaques (*Macaca fascicularis*), aged 43-45 months (7 females and 5 males) and original from Mauritian AAALAC certified breeding centers were used for SARS-CoV-2 challenge studies. All animals were housed in IDMIT facilities (CEA, Fontenay-aux-roses), under BSL-3 containment (Animal facility authorization #D92-032-02, Préfecture des Hauts de Seine, France) and in compliance with European Directive 2010/63/EU, the French regulations and the Standards for Human Care and Use of Laboratory Animals, of the Office for Laboratory Animal Welfare (OLAW, assurance number #A5826-01, US). The protocols were approved by the institutional ethical committee “Comité d’Ethique en Expérimentation Animale du Commissariat à l’Energie Atomique et aux Energies Alternatives” (CEtEA #44) under statement number A20-037. The study was authorized by the “Research, Innovation and Education Ministry” under registration number APAFIS#24434-2020030216 532863.

Cynomolgus macaques were randomly assigned to the two experimental groups. The vaccinated group (n = 6) received a 10^12^ gc of AC1 vaccine candidate while control animals (n = 6) received only the diluent. Blood samples were harvested on weeks 0, 1, 2, 4, 5, 6, 7, 8 and 9. Sixty-seven days after immunization, all animals were exposed to a total dose of 10^5^ pfu of SARS-CoV-2 virus (hCoV-19/France/ lDF0372/2020 strain; GISAID EpiCoV platform under accession number EPI_ISL_406596) via the combination of intranasal and intra-tracheal routes (0.25 mL in each nostril and 4.5 mL in the trachea, i.e. a total of 5 mL; day 0 of challenge), using atropine (0.04 mg/kg) for pre-medication and ketamine (5 mg/kg) with medetomidine (0.05 mg/kg) for anesthesia. Nasopharyngeal and tracheal swabs were collected at 2, 3, 4, 5, 8, 11, 14 and 25 days post exposure (d.p.e.) while blood was taken at 2, 3, 4, 5, 8, 11, 14, 25 and 31 d.p.e. Bronchoalveolar lavages (BAL) were performed using 50 mL sterile saline at 3 and 11 d.p.e. Blood cell counts, hemoglobin and hematocrit were determined from EDTA blood using a DXH800 analyzer (Beckman Coulter).

#### Human samples

Blood was collected from 60 patients with nasopharyngeal PCR-confirmed SARS-CoV-2 infection stratified by disease severity. Plasma was separated and stored at negative 80°C until assessed. Human subject investigation was approved by the institutional Review Board of the Massachusetts General Hospital. Age, sex, and gender identity are unknown.

### Method details

#### Vaccine candidates

Two AAV-based vaccine candidates were tested: AAVCOVID19-1 (AC1) and AAVCOVID19-3 (AC3) (Figure 1A) (GenBank: MW408785 and GenBank: MW408786, respectively). AC1 is an AAVrh32.33 vector that expresses the codon optimized, pre-fusion stabilized (furin cleavage site mutated to G_682_SAS_685_ and P_986_P_987_ substitutions) full length SARS-CoV-2 Spike protein under the control of an SV40 promoter. AC1 carries a short SV40 polyadenylation signal (poly-A). AC3 is an AAVrh32.33 that carries the secreted S1 subunit of SARS-CoV-2 Spike with the tissue plasminogen activator signal peptide (tPA-SP) whose expression is driven by the CMV promoter. AC3 has two more regulatory elements: a woodchuck hepatitis virus posttranscriptional regulatory element (WPRE) and the bovine growth hormone polyadenylation signal (poly-A).

#### Small-scale production of vaccine candidates

Research-grade, high-titer vectors were produced, purified, and titrated by the MEEI/ SERI Gene Transfer Vector Core (https://www.vdb-lab.org/vector- core/). Small-scale vector preparations were generated by polyethylenimine or PEI (Polysciences, Cat #24765-2) triple transfection of AC1 or AC3 ITR-flanked transgene, pKan2/rh32.33 (AAV2 rep and AAVrh32.33 capsid construct), and pALD-X80 adenoviral helper plasmid in a 1:1:2 ratio, respectively, in HEK293 cells. DNA was transfected in 10-layer HYPERFlasks using a PEI-Max/DNA ratio of 1.375:1 (v/w). 3 days after transfection, vectors were harvested from the HYPERFlasks using Benzonase (EMD Millipore, catalog no. 1016970010) to degrade DNA/RNA. 24 hours after harvesting, the vectors were concentrated by tangential flow filtration and purified by iodixanol gradient ultracentrifugation as previously described (Lock et al., 2010). Vaccine candidates were quantified by ddPCR according to a previously published protocol (Sanmiguel et al., 2019). Capsid stability was assessed by AAV-ID (Pacouret et al., 2017).

#### Large-scale manufacturing

AAVCOVID candidates were produced at larger scale via standard AAV production processes by Novartis Gene Therapies, following their stablished protocol with only minimal modifications to adjust to the AAVrh32.33 technology. Briefly, AC1 and AC3 were produced via three plasmid transfection (AC1 or AC3 ITR-flanked transgene, pKan2/rh32.33 (AAV2 rep and AAVrh32.33 capsid construct), and pALD-X80 adenoviral helper plasmid) in an iCellis500 bioreactor (Pall Biosciences). Following cell lysis and lysate clarification, tangential flow filtration (TFF) was conducted to achieve volume reduction. The TFF retentate was next enriched for AAV particles on a cation exchange chromatography column (BIA Separations, Sartorius). The eluate was concentrated, and buffer exchanged through an additional TFF step, before CsCl ultracentrifugation to separate genome containing versus empty AAV particles. Finally, formulation (buffer: 20 mM tris (pH 8.1 ± 0.1), 1 mM magnesium chloride (MgCl2), 200 mM sodium chloride (NaCl) and 0.005% poloxamer 188) was achieved through TFF before bulk drug substance was filtered.

#### SARS-CoV-2 spike TaqMan assay

The codon optimized SARS-CoV-2 receptor binding domain (RBD) of AAVCOVID vaccine candidates was used as a target for droplet digital PCR (ddPCR)/real-time PCR (qPCR) quantifications. The sequence was checked for secondary structures using the mfold application of the UNAfold software package (Zuker, 2003) at the PCR annealing temperature and TaqMan buffer salt concentrations.Internal repeats were avoided by maping against the entire codon optimized SARS-CoV-2 S gene of AAVCOVID candidates using the REPuter application (Kurtz et al., 2001). The 5’-end of the gene was selected as PCR target based on these analyses. The oligo sequences used were the following: forward primer, GTGCAGCCAACCGAG (0.43μM final concentration); reverse primer, ACACCTCGCCAAATGG (1.125μM final concentration), and TaqMan® probe 6FAM- TCTATCGTGCGCTTTC-MGBNFQ (0.25μM final concentration). The final concentration and Tm’s of primers were determined using the DINAMelt application of the UNAfold software package (Markham and Zuker, 2005, 2008) and set to hybridize the target with a Tm of just under 60°C (59.0-59.9°C) for high specificity. The PrimerExpress™ software (Applied Biosystems™) was used to determine the Tm of the MGB probe (Kutyavin et al., 2000). The resulting 67 bp amplicon was inspected for specificity via NCBI BLAST® using the somewhat similar algorithm in the suite against human, NHP, mouse, ferret, and Betacoronavirus databases and determined to be highly specific for our vaccine candidates. No significant matches were found against the RBD oligonucleotides used.

#### *In vitro* expression studies

10^5^ HEK293 cell/well were seeded in 12-well plates (Corning, MA, USA) plates and incubated at 37°C overnight. The following day, cells were transfected with 2 μg of AAVCOVID19-1 (pAC1) and AAVCOVID19-3 (pAC3) plasmids using PEI-Max. Cells were harvested 24 and 72 hours after transfection for mRNA and western blot (WB) expression analyses, respectively. In addition, 5x10^4^ HuH7 cell/well were seeded in 12-well plates and incubated overnight at 37°C. On the following day, adenovirus 5 WT (Ad5) was added to the cells at a MOI of 20 pfu/cell. 2 hours later, media was removed and cells infected with a MOI of 5x10^5^ of AC1 or AC3. Cells were harvested 72 hr later for WB analysis.

Transfection and transduction samples were also collected for RNA gene expression analyses. Total RNA was extracted via Trizol™ reagent (Invitrogen™) and quantified using a Qubit™ fluorometer (Invitrogen™). 7.5μg of Total RNA was DNase-I treated using the Turbo DNA-free™ kit (Invitrogen™). About 1.4μg of DNase-treated total RNA was set aside for reverse transcription against (-)RT controls using the high capacity cDNA reverse transcription kit (Thermo Fisher™). Codon optimized RBD gene expression was assessed against a cells only control using qPCR and normalized to human 18S rRNA gene levels by the delta delta Ct method (Livak and Schmittgen, 2001).

#### Detection of spike antigens by western blot

Cell lysates were obtained by diluting cell pellets in NuPAGE™ LDS Sample Buffer (4X) (Thermo Fisher Scientific, Cat# NP0007) and incubating at 99°C for 5 minutes, separated by electrophoresis in NuPAGE 4-12% polyacrylamide gels (Thermo Fisher Scientific, Cat#NP0321PK2) and then transferred to PVDF membranes. The membranes were probed with an anti-SARS-CoV-2 RBD rabbit polyclonal antibody (Sino Biological Inc., 40592-T62) followed by a goat anti-rabbit HRP-conjugated secondary antibody (Thermo Fisher Scientific, Cat# A16110, RRID AB_2534782). Membranes were developed by chemiluminescence using the Immobilon Western Chemiluminescent HRP Substrate (Millipore, Cat# WBKLS0500) and recorded using ChemiDoc MP Imaging System (Bio-Rad). An anti-GAPDH antibody (Cell Signaling Technology Cat# 2118, RRID:AB_561053) was used as loading control.

#### SARS-CoV-2 binding antibody ELISA

Nunc MaxiSorp™ high protein-binding capacity 96 well plates (Thermo Fisher Scientific, Cat# 44-2404-21) were coated overnight at 4 °C with 1μg/ml SARS-CoV-2 RBD diluted in phosphate-buffered saline (PBS). The next day the plates were washed with PBS-Tween 20 0.05% (Sigma, Cat# P2287-100ML) using the Biotek 405 TS Microplate washer. Each plate was washed five times with 200 μl wash buffer and then dried before the next step. Following the first wash, 200 μl of Blocker Casein in PBS (Thermo Fisher Scientific, Cat# 37528) were added to each well and incubated for 2 hours at RT. After blocking, serum samples were serially diluted in blocking solution starting into 1:100 dilution. Rhesus BAL samples were added undiluted and serially diluted in blocking solution. After an hour of incubation, the plates were washed and 100 μl of secondary Peroxidase AffiniPure Rabbit Anti-Mouse IgG (Jackson ImmunoResearch, Cat# 315-035-045, RRID: AB_2340066) antibody diluted 1:1,000 in blocking solution, rabbit Anti-Monkey IgG (whole molecule)-Peroxidase antibody (Sigma-Aldrich Cat# A2054, RRID:AB_257967) or anti-Monkey IgA (NIH Nonhuman Primate Reagent Resource supported by AI126683 and OD 010976) diluted 1:5,000 were added to each well. After one hour of incubation at room temperature, the plates were washed and developed for 3.5 min with 100 μl of Seracare SureBlue Reserve™ TMB Microwell Peroxidase Substrate solution (SeraCare, Cat# 53-00-03). The reaction was then stopped with 100 μl Seracare KPL TMB Stop Solution (SeraCare, Cat# 50-85-06). Optical density (OD) at 450 nm was measured using a Biotek Synergy H1 plate reader. The titer was the reciprocal of the highest dilution with absorbance values higher than four times the average of the negative control wells. For mouse serum SARS-CoV-2 RBD-specific antibody isotyping, the same ELISA was performed but using the secondary antibodies from SBA Clonotyping System-HRP kit (SouthernBiotech, 5300-05, RRID:AB_2796080) diluted accordingly to manufacturer’s instructions.

Cynomolgus macaque samples were screened for IgG to SARS-CoV-2 antigens using a commercially available multiplexed immunoassay developed by Mesoscale Discovery (MSD,Rockville, MD) as previously described (Johnson et al., 2020b). Briefly, antigens were spotted at 200−400 μg/mL in a proprietary buffer, washed, dried and packaged for further use (MSD® SARS-CoV2 Plate 2). Then, plates were blocked with MSD Blocker A and then incubated with reference standard, controls and samples diluted 1:500 and 1:5000 in diluent buffer. After incubation, a detection antibody was added (MSD SULFO-TAGTM Anti-Human IgG Antibody) followed by the MSD GOLDTM Read Buffer B. Plates were read using a MESO QuickPlex SQ 120MM Reader. Results were expressed as arbitrary units (AU)/ml.

The MSD pseudo-neutralization assay was used to measure antibodies neutralizing the binding of the spike protein to the ACE2 receptor in cynomolgus samples. Plates were blocked and washed as above, assay calibrator (COVID- 19 neutralizing antibody; monoclonal antibody against S protein; 200 μg/mL), control sera and test sera samples diluted 1:10 and 1:100 in assay diluent were added to the plates. Following incubation of the plates, a 0.25 μg/mL solution of MSD SULFO-TAGTM conjugated ACE-2 was added and the plates were read as above. Electro-chemioluminescence (ECL) signal was recorded and results expressed as 1/ECL.

#### *Pseudovirus* production and titration

Lenti-SARS2 was produced based on a published protocol (Crawford et al., 2020). Specifically, 50% confluent HEK293T cells were seeded 24 hours prior to transfection in 15 cm plates. The next day, 18 μg/plate of psPAX2, 9 μg/plate of pCMV-SARS2-Spike (WT or VOC) and 29 μg/plate of pCMV-Lenti-Luc plasmids were mixed in 3.6 mL/plate of Opti-MEM™ I Reduced Serum media (Gibco, Cat# 31985070) along with 144 μL of PEI Max 40K (1 mg/mL, pH: 6.9-7.1) and mixed thoroughly. The mixture was incubated for 20 minutes at room temperature. Media on cells was aspirated and serum-free DMEM was added to the cells. After 20 mins, the DNA-PEI mixture was added dropwise to the plate and incubated overnight at 37°C with 5% CO_2_. The next day, media was replaced with DMEM with 10% FBS. After 48 hours, the media was collected in a 50 mL conical and centrifuged at 4,000 rpm at 4°C for 5 minutes to remove cell debris. The supernatant was collected and filtered through 0.45 μm filter, aliquoted and stored at -80°C.

For titration of the pseudovirus, HEK293T cells expressing ACE2 were seeded at 1.5x10^4^ cells/well in poly-L-Lysine (0.01%) coated 96-well black plates (Thermo Fisher Scientific, Cat# 3904) one day before titration. On the next day, the media was changed to 50 μL DMEM+10%FBS containing filtered Hexadimethrine bromide at a final concentration of 10 μg/mL. 2-fold serial dilutions (up-to 15 dilutions) of the viral stocks (50μL) were added to the plate in 6 replicates each and incubated for 48 hours. After 48 hours, cells were lysed with Reporter Lysis Buffer (Promega, Cat# E4030). These plates were frozen at -80°C for 60 minutes. Thereafter, they were thawed at 37°C for 20 mins before starting the luciferase readout. For luciferase substrate buffer the following reagents were mixed; Tris-HCl buffer at 0.5 M, ATP at 0.3 mM, MgCl2 at 10 mM, Pierce™ Firefly Signal Enhancer (Thermo Fisher Scientific, Cat#16180), D-luciferin 150μg/mL (PerkinElmer, Cat# 122799). Biotek Synergy H1 Plate reader was used for luminescence readout. For pseudovirus neutralization assay, a final dilution of the virus stock targeting relative luminescence units (RLU) of 1800-1100 was used which was approximately 200-fold higher than background signal obtained in untreated cells.

#### *Pseudovirus* neutralization assay

HEK293T cells expressing ACE2 were seeded at 1.5x10^4^ cells/well in poly-L-Lysine (0.01%) coated 96-well black plates. The following day, 50μL of DMEM+10% FBS media containing Hexadimethrine bromide (final concentration 10 μg/mL) was added to the cells. Serum samples were heat-inactivated at 56°C for 1 hour. Serum samples were then serially diluted (2-fold) for 10 dilutions in DMEM with 10% FBS with initial dilution of 1:40 for mouse serum and 1:10 dilution for NHP serum. Thereafter, Lenti-SARS2 pseudovirus was added to each dilution and incubated at 37°C for 45 minuntes. The serum and virus mixture was added to the cells and incubated at 37°C with 5% CO2 for 48 hours. An anti-SARS-CoV-2 Spike monoclonal neutralizing antibody (GenScript, Cat# A02055) was used as a positive control. Cells without serum and virus were used as negative control. After 48 hours, cells were lysed and luciferase measured as described above. Neutralizing antibody titers or 50% inhibitory concentration in the serum sample (EC50 or ID50) were calculated as the reciprocal of the highest dilution showing less RLU signal than half of the average RLU (maximum infectivity) of Virus Control group (cells + virus, without serum). Pseudovirus neutralizing titers were converted to international units (IU)/mL with the NIBSC 20/136 WHO International Standard.

#### Plaque Reduction Neutralization Test (PRNT)

Depending on the volume available, mouse or NHP sera were serially diluted two-fold from an initial dilution of either 1:12.5 or 1:25 for ten dilutions in Dulbecco’s Phosphate Buffered Saline (DPBS, Gibco). Each dilution was incubated at 37°C and 5% CO2 for 1 hour with an equal volume of 1000 plaque forming units/ml (PFU/ml) of SARS-CoV-2 (isolate USA-WA1/2020) diluted in DMEM (Gibco) containing 2% fetal bovine serum (Gibco) and antibiotic-antimycotic (Gibco). Controls included DMEM containing 2% fetal bovine serum (Gibco) and antibiotic-antimycotic (Gibco) only as a negative control, 1000 PFU/ml SARS-CoV-2 incubated with DPBS, and 1000 PFU/ml SARS-CoV-2 incubated with DMEM. Two hundred microliters of each dilution or control were added to confluent monolayers of NR-596 Vero E6 cells in triplicate and incubated for 1 hour at 37°C and 5% CO2. The plates were gently rocked every 5-10 minutes to prevent monolayer drying. The monolayers were then overlaid with a 1:1 mixture of 2.5% Avicel® RC-591 microcrystalline cellulose and carboxymethylcellulose sodium (DuPont Nutrition & Biosciences) and 2X Modified Eagle Medium (Temin’s modification, Gibco) supplemented with 2X antibiotic-antimycotic (Gibco), 2X GlutaMAX (Gibco) and 10% fetal bovine serum (Gibco). Plates were incubated at 37°C and 5% CO2 for 2 days. The monolayers were fixed with 10% neutral buffered formalin and stained with 0.2% aqueous Gentian Violet (RICCA Chemicals) in 10% neutral buffered formalin for 30 minutes, followed by rinsing and plaque counting. The half maximal inhibitory concentrations (EC50 or ID50) were calculated using GraphPad Prism 8.

#### IFN-γ and IL-4 ELISPOT assay in mouse

Splenocytes were obtained by grinding murine spleens with 100 μm cell strainers, followed by treatment with Ammonium Chloride-Potassium (ACK) lysis buffer (Gibco) to lyse the red blood cells. The isolated cells were then suspended in complete RPMI-1640 medium (Gibco) supplemented with 10% FBS and counted for the following experiments.

IFN-γ and IL-4 ELISPOT for mice was measured as previously described (Wang et al., 2019). Briefly, 96-well PVDF plates (Millipore) were pre-coated with 10 μg/ml anti-mouse IFN-γ ELISPOT capture antibody (BD Biosciences Cat# 551881, RRID:AB_2868948) or 4 μg/ml anti-mouse IL-4 ELISPOT capture antibody (BD Biosciences Cat# 551878, RRID:AB_2336921) at 4°C overnight, and then blocked with complete RPMI-1640 medium for 3 hours at 37°C. One million of freshly isolated splenocytes were seeded into the precoated plates and stimulated with S1 and S2 peptides pools (GenScript) with a final concentration of 1 μg/ml of each peptide diluted in RPMI-1640 supplemented with 10% FBS and incubated for 48 hours at 37°C with 5% CO_2_. Each peptide pool, consisting of 15-mers peptides overlapping by 10 amino acids, spanning the entire SARS-CoV-2 Spike protein S1 or S2 subunits. Control wells contained 5x10^5^ cell stimulated with DMSO diluted in RPMI-1640 supplemented with 10% FBS (negative control) or 2 μg/ml concanavalin A (positive control). Subsequently, the plates were washed and incubated with biotin-conjugated mouse IFN-γ ELISPOT Detection Antibody (BD Biosciences Cat# 551881, RRID:AB_2868948) and 4 μg/ml biotin-conjugated mouse IL-4 detection antibody (BD Biosciences Cat# 551878, RRID:AB_2336921) at room temperature for 3 hours and followed by streptavidin-HRP (dilution 1:1000, Sigma-Aldrich, Cat# 18-152) for 45 minutes. After washing, 100 μL/well of NBT/BCIP substrate solution (Promega, Cat# S3771) were added and developed for 15–30 min until distinct spots emerged. The cytokine-secreting cell spots were imaged and counted on AID EliSpot reader (Autoimmun Diagnostika GmbH).

#### IFN-γ ELISPOT assay in NHP PBMCs

Rhesus macaque peripheral blood T cell responses against AC1, AC3 and the AAVrh32.33 capsid were measured by interferon gamma (IFN-γ) enzyme-linked immunosorbent spot (ELISPOT) assays according to previously published methods (Calcedo et al., 2018). Peptide libraries specific for AAVrh32.33 capsid as well as the AC1 and AC3 transgenes were generated (15-mers with a 10 amino acid overlap with the preceding peptide; Mimotopes, Australia). Peptides were dissolved in dimethyl sulfoxide (DMSO) at a concentration of 100 mg/mL, pooled, aliquoted and stored at -80°C. They were used at a final concentration in the assay of approximately 2 μg/mL. The positive response criteria for the IFN-γ ELISPOT was greater than 55 spot forming units (SFU) per million cells and at least three times greater than the negative control values.

IFNγ ELISpot assay was performed in cynomolgus macaque PBMCs using the Monkey IFNg ELISpot PRO kit (Mabtech, #3421M-2APT) according to the manufacturer’s instructions. PBMCs were plated at a concentration of 200,000 cells per well and were stimulated with SARS-CoV-2 peptids (PepMixTM) synthethized by JPT Peptide Technologies (Berlin, Germany). These 15 mer peptids are divided in two pools (S1 and S2) of respectively 158 and 157 peptids overlapping by 11 amino acids. The peptids are coding for the S protein of SARS-CoV-2 and will be used at a final concentration of 2 μg/mL. Plates were incubated for 18 h at 37C in an atmosphere containing 5% CO_2_, then washed 5 times with PBS and incubated for 2 h at 37C with a biotinylated anti-IFNγ antibody. After 5 washes, spots were developed by adding 0.45 mm-filtered ready-to-use BCIP/NBT-plus substrate solution and counted with an automated ELISpot reader ELRIFL04 (Autoimmun Diagnostika GmbH, Strassberg, Germany). Spot forming units (SFU) per 10^6^ PBMCs are means of duplicates for each animal.

#### Titration of antibodies in BAL samples

RBD-binding IgG and IgA and pseudovirus neutralizing antibody levels were corrected for BAL by quantifying serum and BAL urea levels using the Urea Assay Kit (abcam, ab83362) as described previously (Kaulbach et al., 1993).

#### PBMC stimulation and flow cytometry

Cryopreserved peripheral blood mononuclear cells (PBMC) were thawed and rested overnight in sterile R10 media (RPMI 1640, Corning), supplemented with 10% fetal bovine serum (Gemini Bio-Products), Penicillin/Streptomycin and L-Glutamine; plus 10 U/mL DNAse I (Roche Life Sciences) at 37°C, 5% CO_2_ and 95% humidity incubation conditions. PBMC were stimulated at 200 ul final volume in sterile R10 media. Peptide concentrations for stimulation conditions were 2 μg/ml for AC1/AC3 shared peptide pool A, B and C and AAVrh32.22 peptide pool A, B and C. Co-stimulation was added with peptides: 1μg/mL anti-CD49d (Clone 9F10, BioLegend Cat# 304301, RRID:AB_314427) and CD28-ECD (Clone CD28.2, Beckman Coulter Cat# 6607111, RRID:AB_1575955) at the start of stimulation. Positive control samples were stimulated using Staphylococcal Enterotoxin B (SEB, List Biological Laboratories) at 1μg/mL. CD107a BV650 (clone H4A3, BioLegend Cat# 328643, RRID:AB_2565967) was added at the start of stimulation. Brefeldin A (1μg /mL) (Sigma-Aldrich) and monensin (0.66μL/mL) (BD Biosciences) were added one hour after initiation of stimulation. Cells were incubated under stimulation conditions for a total of 9 hours.

All following incubations were performed at room temperature. Cells were stained for viability exclusion using Live/Dead Fixable Aqua for 10 minutes, followed by a 20-minute incubation with a panel of directly conjugated monoclonal antibodies diluted in equal parts of fluorescence-activated cell sorting (FACS) buffer (PBS containing 0.1% sodium azide and 1% bovine serum albumin) and Brilliant stain buffer (BD Biosciences). Fluorophore-conjugated recombinant RBD protein produced by the Hensley Lab (University of Pennsylvania) was used to identify RBD-binding B cells during the surface antibody stain. Cells were washed in FACS buffer and fixed/permeabilized using the FoxP3 Transcription Factor Buffer Kit (eBioscience), following manufacturer’s instructions. Intracellular staining was performed by adding the antibody cocktail prepared in 1X permwash buffer for 1 hour. Stained cells were washed and fixed in PBS containing 1% paraformaldehyde (Sigma-Aldrich) and stored at 4°C in the dark until acquisition. All flow cytometry data were collected on a BD LSR II or BD FACSymphony A5 cytometer (BD Biosciences). Data were analyzed using FlowJo software (versions 9.9.6 and 10.6.2, Tree Star).

The following antibodies were used: PD1 BV421 (clone EH12.2H7, BioLegend Cat# 329919, RRID:AB_10900818), CD14 BV510 (clone M5E2, BioLegend Cat# 301842, RRID:AB_2561946) and APC-Cy7 (clone M5E2, BioLegend Cat# 301819, RRID:AB_493694), CD16 BV510 (clone 3G8, BioLegend Cat# 302048, RRID:AB_2562085) and APC-Cy7 (clone 3G8, BioLegend Cat# 302017, RRID:AB_314217), CD20 BV510 (clone 2H7, BioLegend Cat# 302339, RRID:AB_2561721) and BV650 (clone 2H7, BioLegend Cat# 302335, RRID:AB_11218609), CD69 BV605 (clone FN50, BioLegend Cat# 310937, RRID:AB_2562306), CD21 PECy7 (clone Bu32, BioLegend Cat# 354911, RRID:AB_2561576), CD4 BUV661 (clone SK3, BD Biosciences Cat# 612962, RRID:AB_2870238), CD95 BUV737 (clone DX2, BD Biosciences Cat# 612790, RRID:AB_2870117), CD8 BUV563 (clone RPA-T8, BD Biosciences Cat# 612914, RRID:AB_2870199), KI67 BV786 (clone B56, BD Biosciences Cat# 563756, RRID:AB_2732007), IL2 PE (clone MQ1-17H12, BD Biosciences Cat# 554566, RRID:AB_395483), IFNγ BV750 (clone B27, BD Biosciences Cat# 566357, RRID:AB_2739707), CD3 BUV805 (clone SP34-2, BD Biosciences Cat# 742053, RRID:AB_2871342), Granzyme B AF700 (clone GB11, BD Biosciences Cat# 560213, RRID:AB_1645453), CD3 APC-Cy7 (clone SP34-2, BD Biosciences Cat# 557757, RRID:AB_396863), IgM PECy5 (clone G20-127, BD Biosciences Cat# 551079, RRID:AB_394036), CD27 BV421 (clone M-T271, BD Biosciences Cat# 562513, RRID:AB_11153497), HLA-DR BV605 (clone G46-6, BD Biosciences Cat# 562844, RRID:AB_2744478), CD80 BV786 (clone L307.4, BD Biosciences Cat# 564159, RRID:AB_2738631), CXCR3 AF488 (clone 1C6, BD Biosciences Cat# 558047, RRID:AB_397008), CXCR5 SB702 (clone MU5BEE, Thermo Fisher Scientific Cat# 67-9185-42, RRID:AB_2717183), Tbet PerCP-Cy5.5 (clone 4B10, Thermo Fisher Scientific Cat# 45-5825-82, RRID:AB_953657), CD11c PECy5.5 (clone 3.9, Thermo Fisher Scientific Cat# 35-0116-42, RRID:AB_11218511), TNFα PE-Cy7 (clone Mab11, Thermo Fisher Scientific Cat# 25-7349-41, RRID:AB_1257208), and polyclonal anti-IgD PE Tx Red (SouthernBiotech Cat# 2030-09, RRID:AB_2795630).

First, to ensure that only live single cells were analyzed from PBMCs, forward scatter height (FSC-H)-versus-forward scatter area (FSC-A) and side scatter area (SSC-A)-versus-FSC-A plots were used to exclude doublets and focus on singlet small lymphocytes. Dead cells were excluded by gating on cells negative for the viability marker Aqua Blue. For T cell function analysis, monocytes, B cells and NK cells were excluded via the CD14/19/16 dump gate. CD4^+^ and CD8^+^ T lymphocytes were gated within CD3^+^ cells. To determine the memory phenotype, CD28 versus CD95 were used, and naïve T cells were excluded from the analysis.

To track vaccine-induced peripheral blood B cells, a double-labeling technique with fluorophore-conjugated SARS-CoV-2 recombinant RBD protein was utilized. Analysis of RBD-binding memory B cells (MBCs) (Figure S4A) (Johnson et al., 2020a; Knox et al., 2017). For B cell analysis, B cells were identified as CD20^+^ and CD3/CD14/CD16^-^. Memory B cells were defined as CD27^+^ or CD27^-^IgD^-^.

#### SARS-CoV-2 genomic and subgenomic RNA RT-qPCR

Upper respiratory (nasopharyngeal and tracheal) specimens were collected with swabs (Viral Transport Medium, CDC, DSR-052-01). Tracheal swabs were performed by insertion of the swab above the tip of the epiglottis into the upper trachea at approximately 1.5 cm of the epiglottis. All specimens were stored between 2°C and 8°C until analysis by RT-qPCR with a plasmid standard concentration range containing an RdRp gene fragment including the RdRp-IP4 RT-PCR target sequence. The limit of detection was estimated to be 2.67 log_10_ copies of SARS-CoV-2 gRNA per mL and the limit of quantification was estimated to be 3.67 log_10_ copies per mL. SARS-CoV-2 E gene subgenomic mRNA (sgRNA) levels were assessed by RT-qPCR using primers and probes previously described (Corman et al., 2020; Wölfel et al., 2020): leader-specific primer sgLeadSARSCoV2-F CGATCTCTTGTAGATCTGTTCTC, E-Sarbeco-R primer ATATTGCAGCAGTACGCACACA and E-Sarbeco probe HEX-ACACTAGCCATCCTTACTGCGCTTCG-BHQ1. The protocol describing the procedure for the detection of SARS-CoV-2 is available on the WHO website (https://www.who.int/docs/default-source/coronaviruse/real-time-rt-pcr-assays-for-the-detection-of-sars-cov-2-institut-pasteur-paris.pdf?sfvrsn=3662fcb6_2). The limit of detection was estimated to be 2.87 log_10_ copies of SARS-CoV-2 sgRNA per mL and the limit of quantification was estimated to be 3.87 log_10_ copies per mL.

#### [^18^F]-FDG PET/CT protocol

All imaging acquisitions were performed on the Digital Photon Counting (DPC) PET-CT system (Vereos-Ingenuity, Philips) (Zhang et al., 2016) implemented in BSL3 laboratory.

For imaging sessions, animals were first anesthetized with Ketamine (10mg/kg) + Metedomidine (0.05mg/kg) and then maintained under isofluorane 2% in a supine position on a patient warming blanket (Bear Hugger, 3M) on the machine bed with cardiac rate, oxygen saturation and temperature monitoring.

CT was performed under breath-hold 5 minutes prior to PET scan for attenuation correction and anatomical localization. The CT detector collimation used was 64 × 0.6 mm, the tube voltage was 120 kV and intensity of about 150mAs. Automatic dose optimization tools (Dose Right, Z-DOM, 3D-DOM by Philips Healthcare) regulated the intensity. CT images were reconstructed with a slice thickness of 1.25 mm and an interval of 0.25 mm.

A whole-body PET scan (4–5 bed positions, 3 min/bed position) was performed 45 min post injection of 3.39±0.28 MBq/kg of [^18^F]-FDG via the saphenous vein. PET images were reconstructed onto a 256 x 256 matrix (3 iterations, 17 subsets).

Images were analyzed using INTELLISPACE PORTAL 8 (Philips healthcare) and 3DSlicer (Open source tool). Different regions of interest (lung and lung draining lymph nodes) were defined by CT and PET. Pulmonary lesions were defined as Ground Glass Opacity, Crazy-paving pattern or consolidation as previously described (Maisonnasse et al., 2020; Pan et al., 2020; Shi et al., 2020). Lesion features detected by CT imaging were assessed by two analyzers independently and final CT score results were obtained by consensus.

Besides, regions with FDG uptake (lung, lung draining lymph nodes and spleen) were also defined for quantification of SUV parameters, including SUVmean, SUVmax.

#### AAV neutralizing antibody (NAb) assay

NAb responses against AAV1, AAV2, AAV5, AAV8, AAV9 and AAVrh32.33 capsids were measured in serum using an in vitro HEK293 cell-based assay and LacZ expressing vectors (Vector Core Laboratory, University of Pennsylvania, Philadelphia, PA) as previously described (Calcedo et al., 2018). The NAb titer values are reported as the reciprocal of the highest serum dilution at which AAV transduction is reduced 50% compared to the negative control. The limit of detection of the assay was a 1:5 serum dilution.

#### Biodistribution/gene expression studies

Tissue collection was segregated for genomic DNA (gDNA) or total RNA work by QIASymphony nucleic acid extraction with the aim of filling up 96-well plates of purified material. A small cut of frozen tissue (∼ 20 mg) was used for all extractions with the exception of gDNA purifications from spleen (1-2 mg). Tissues were disrupted and homogenized in QIAGEN Buffer ATL (180 μL) and lysed overnight at 56°C in the presence of QIAGEN Proteinase K (400 μg) for gDNA, or directly in QIAGEN® Buffer RLT-Plus in the presence of 2-mercaptoethanol and a QIAGEN anti-foaming agent for total RNA purification. Tissue lysates for gDNA extraction were treated in advance with QIAGEN RNase A (400 μg), while tissue homogenates for RNA extraction were DNase-I treated *in situ* in the QIASymphony® during the procedure. Nucleic acids were quantified only if necessary, as a troubleshooting measure. Purified gDNA samples were diluted 10-fold and in parallel into Cutsmart -buffered BamHI-HF (New England Biolabs) restriction digestions in the presence of 0.1% Pluronic F-68 (50μL final volume) that ran overnight prior to quantification. Similarly, DNase-I-treated total RNAs were diluted 10-fold into cDNA synthesis reactions (20 μL final volume) with or without reverse transcriptase using the High Capacity cDNA Reverse Transcription Kit (Thermo Fisher™). For ddPCR (gDNA or cDNA) or qPCR (cDNA), 2 μL of processed nucleic acids were used for quantification using Bio-Rad™ or Applied Biosystems™ reagents, respectively, in 20 μL reactions using default amplification parameters without an UNG incubation step. All the studies included negative control (PBS) groups for comparison. The significantly small variance of multiple technical replicates in ddPCR justified the use of a single technical replicate per sample and no less than three biological replicates per group, gender, or time point. coRBD signal for ddPCR and vector biodistribution (gDNA) was multiplexed and normalized against the mouse transferrin receptor (Tfrc) gene TaqMan™ assay using a commercial preparation validated for copy number variation analysis (Thermo Fisher Scientific). Likewise, coRBD signal for ddPCR and gene expression analysis was multiplexed and normalized against the mouse GAPDH gene, also using a commercial preparation of the reference assay (Thermo Fisher Scientific). Target and reference oligonucleotide probes are tagged with different fluorophores at the 5’-end which allows efficient signal stratification. For qPCR, coRBD and mGAPDH TaqMan assays were run separately to minimize competitive PCR multiplexing issues prior to analysis and delta delta Ct normalization (Livak and Schmittgen, 2001). The limit of detection of the assay was 10 copies/reaction, therefore, wells with less than 10 copies were considered negative.

#### Phylogenetic analysis

First, fourteen representative AAV capsid sequences were aligned by Clustal Omega (Sievers and Higgins, 2018). Substitution models and model parameters were statistically compared (120 in total) through ProtTest3 (Darriba et al., 2011; Guindon and Gascuel, 2003), and the Le, Gascuel Model (Le and Gascuel, 2008) was selected based on the Aikake Information Criterion (AIC). Additionally, amino acid frequencies were determined empirically through the alignment (+F parameter) and evolutionary rates among sites were allowed to vary within five categories by modeling variability with a discrete Gamma distribution (+G parameter), again selected through AIC. A maximum-likelihood approach was then used to infer the evolutionary relationships among the included sequences using MEGA X (Kumar et al., 2018) and the resultant phylogeny rooted along the midpoint of the branch between AAV4 and AAV5 for purposes of visualization. A sequence identity matrix was computed, and the resultant table was used to annotate the phylogeny by percent identity.

### Quantification and statistical analysis

GraphPad Prism 8 was used for graph preparation and statistical analysis. Data were represented as mean ± standard deviation (SD). Groups were compared between them by One-way ANOVA and Tukey’s tests in studies with more than two groups and n≥10, and Kruskal Wallis and Dunn’s testes were used if n<10. Two groups were compared between them using Student’s t test (independent samples, n≥10), Mann Whitney’s U (independent samples, n<10) or Wilcoxon Signed Rank test (paired samples, n<10). Pearson’s correlation coefficient was calculated to assess correlation.

## Data Availability

All data reported in this paper will be shared by the lead contact upon request. This paper does not report original code. Any additional information required to reanalyze the data reported in this paper is availablefrom the lead contact upon request.

## Supplemental information

Document S1. Figures S1–S6

Document S2. Article plus supplemental information
