# Supplementary material for: An AAV-based, room-temperature-stable, single-dose COVID-19 vaccine provides durable immunogenicity and protection in non-human primates
Source: Cell Host Microbe. 2021 Sep 8;29(9):1437–1453.e8. doi: 10.1016/j.chom.2021.08.002 (PMC8346325; doi:10.1016/j.chom.2021.08.002)
Supplement: Document S1. Figures S1–S6 [file mmc1.pdf]

Cell Host & Microbe, Volume *29*

Supplemental information

An AAV-based, room-temperature-stable, single-dose COVID-19 vaccine provides durable immunogenicity and protection in non-human primates

Nerea Zabaleta, Wenlong Dai, Urja Bhatt, Cécile Hérate, Pauline Maisonnasse, Jessica A. Chichester, Julio Sanmiguel, Reynette Estelien, Kristofer T. Michalson, Cheikh Diop, Dawid Maciorowski, Nathalie Dereuddre-Bosquet, Mariangela Cavarelli, Anne-Sophie Gallouët, Thibaut Naninck, Nidhal Kahlaoui, Julien Lemaitre, Wenbin Qi, Elissa Hudspeth, Allison Cucalon, Cecilia D. Dyer, M. Betina Pampena, James J. Knox, Regina C. LaRocque, Richelle C. Charles, Dan Li, Maya Kim, Abigail Sheridan, Nadia Storm, Rebecca I. Johnson, Jared Feldman, Blake M. Hauser, Vanessa Contreras, Romain Marlin, Raphaël Ho Tsong Fang, Catherine Chapon, Sylvie van der Werf, Eric Zinn, Aisling Ryan, Dione T. Kobayashi, Ruchi Chauhan, Marion McGlynn, Edward T. Ryan, Aaron G. Schmidt, Brian Price, Anna Honko, Anthony Griffiths, Sam Yaghmour, Robert Hodge, Michael R. Betts, Mason W. Freeman, James M. Wilson, Roger Le Grand, and Luk H. Vandenberghe

Figure S1

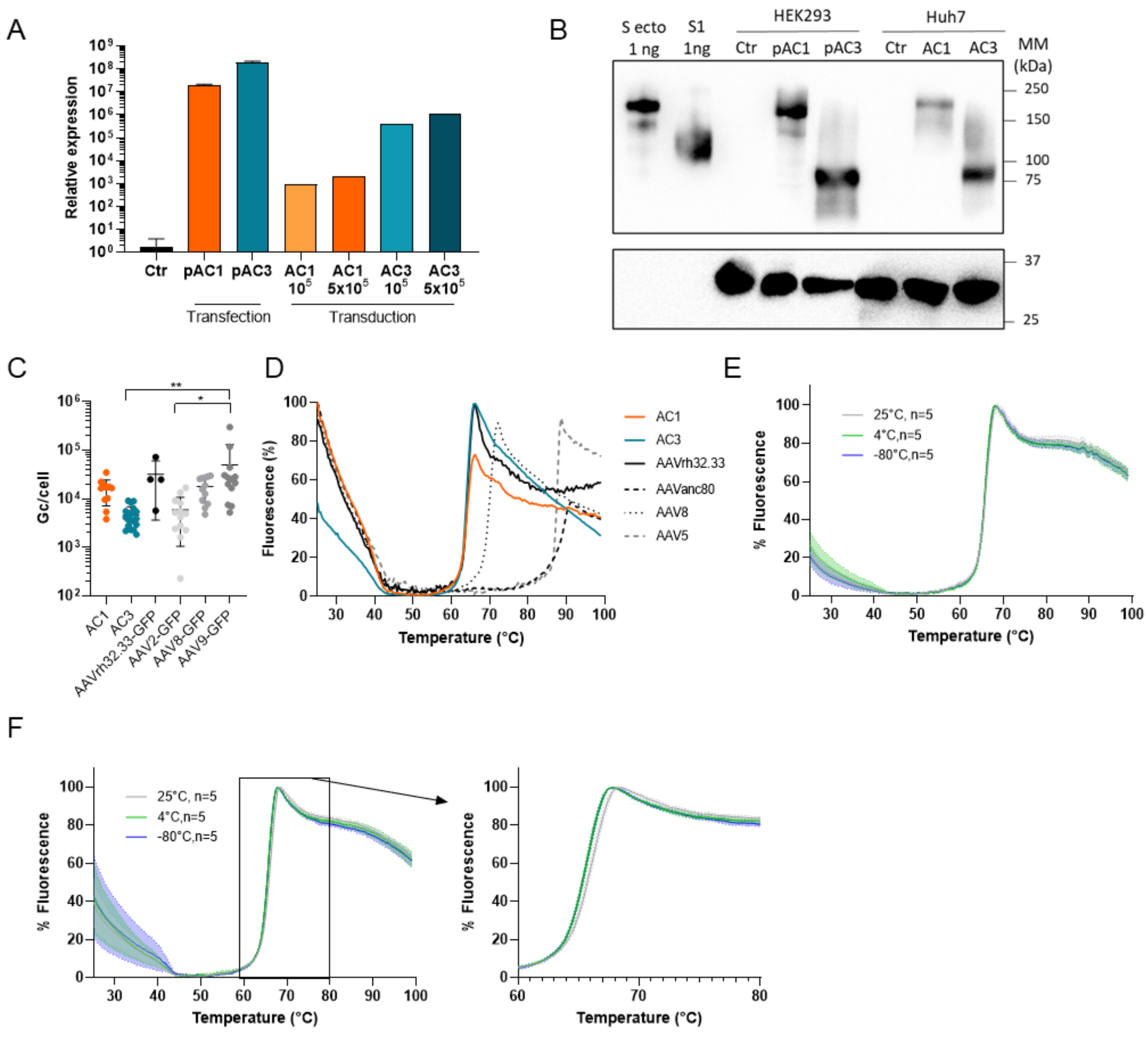

Figure S1. Related to Figure 1. Productivity and in vitro expression of AAVCOVID vaccine candidates

(A) coRBD mRNA expression relative to human 18S rRNA in HEK293 cells transfected with 1 µg of the ITR-containing pAC1 or pAC3 plasmids or transduced with $1x10^5$ or $5x10^5$ gc/cell of AC1 or AC3 24h after treatment. Ctr: untreated cells.

(B) Detection of SARS-CoV-2 Spike antigens by Western blot in HEK293 cells transfected with 1 µg of ITR-containing pAC1 or pAC3 plasmids and Huh7 cells transduced with $5x10^5$ gc/cell of AC1 and AC3 72h after treatment. Recombinant S ectodomain (S ecto, lane 1) and S1 subunit (S1, His-tagged, lane 2) were used as positive control and size reference.

(C) Productivity of several AC1 and AC3 (vector genome copies produced per producer cell or Gc/cell) compared to various AAV serotypes carrying a CMV-EGFP-WPRE transgene in small scale production and purification. Data are represented as mean ± SD. One-way ANOVA and Tukey's tests were used to compare groups between them. * $p<0.05$, ** $p<0.01$.
(D) AAV-ID analysis of capsid identity and stability of AC1 and AC3 compared to AAVrh32.33 and other serotypes.
(E-F) AAV-ID analysis of capsid identity in AC1 aliquots (n=5) stored at -80ºC, 4ºC and 25ºC for 4 (E) and 12 (F) weeks. Data are represented as mean ± SD.

Figure S2

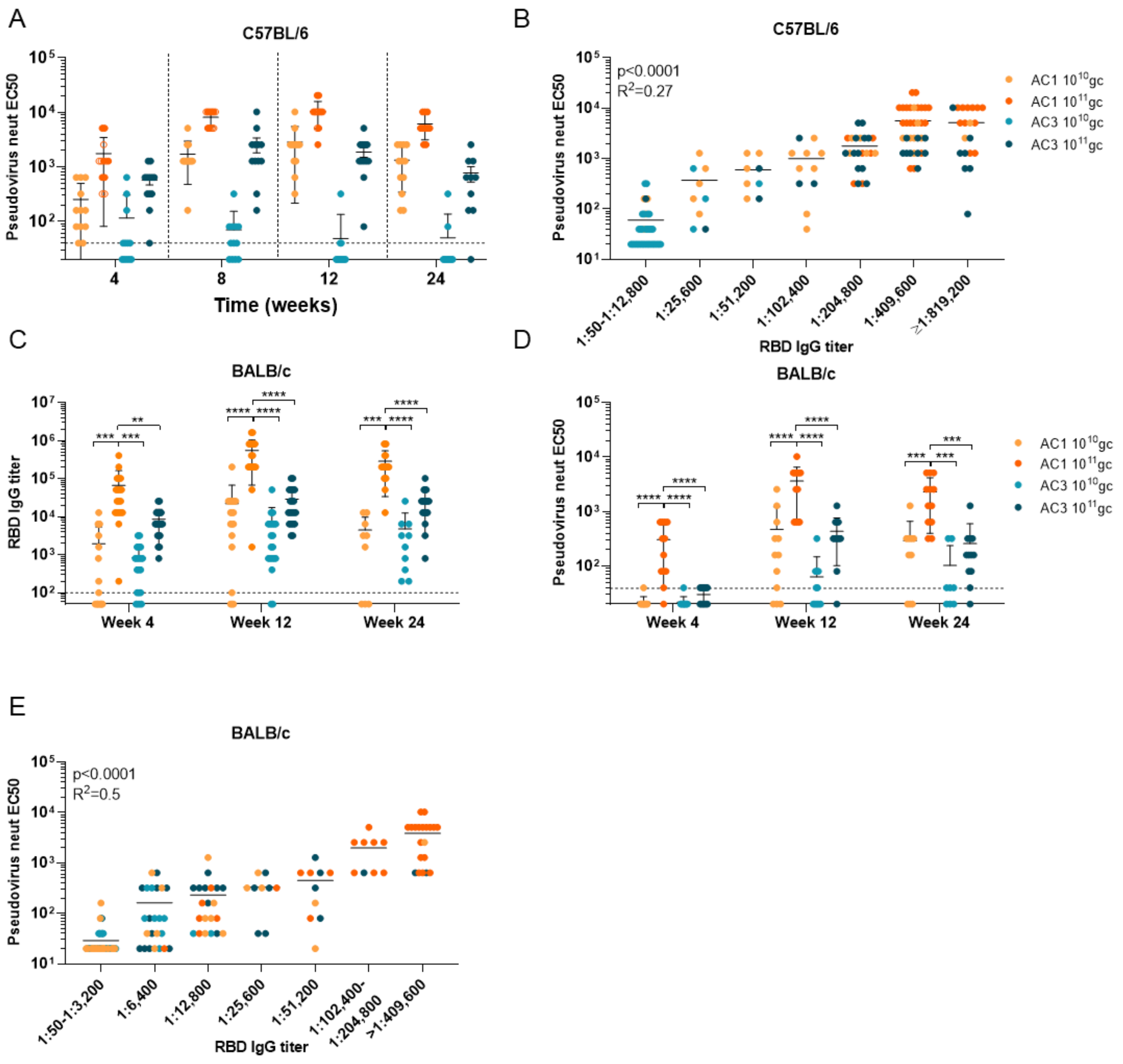

Figure S2. Related to Figure 2. Humoral immunogenicity in BALB/c mice

(A) Monthly monitoring of RBD-binding IgG titers in 7-8 week-old C57BL/6 mice injected IM with two doses (10^{10} gc and 10^{11} gc) of AC1 or AC3, n=20 (10 females and 10 males). The dotted line indicates the lower detection limit of the assay.

(B) Correlation of pseudovirus neutralizing titers and RBD-binding IgG titers in C57BL/6 mice.

(C) Monitoring of SARS-CoV-2 RBD-binding IgG titers in 8-9 week-old BALB/c mice injected IM with two doses (10^{10} gc and 10^{11} gc) of AC1 or AC3, n=20 (10 females and 10 males). The dotted line indicates the lower detection limit of the assay.

(D) Pseudovirus neutralizing titers (international units (IU)/mL) of a subset of BALB/c animals (6 females and 6 males per group) from the study described in A. The dotted line indicates the lower detection limit of the assay.
(E) Correlation of pseudovirus neutralizing titers and RBD-binding IgG titers in BALB/c.
All data are represented as mean ± SD. For (C and D) groups were compared by one-way ANOVA and Tukey's post-test. For (B and E) Pearson's correlation coefficient was calculated to assess correlation.
* $p<0.05$, ** $p<0.01$, *** $p<0.001$, **** $p<0.0001$.

Figure S3

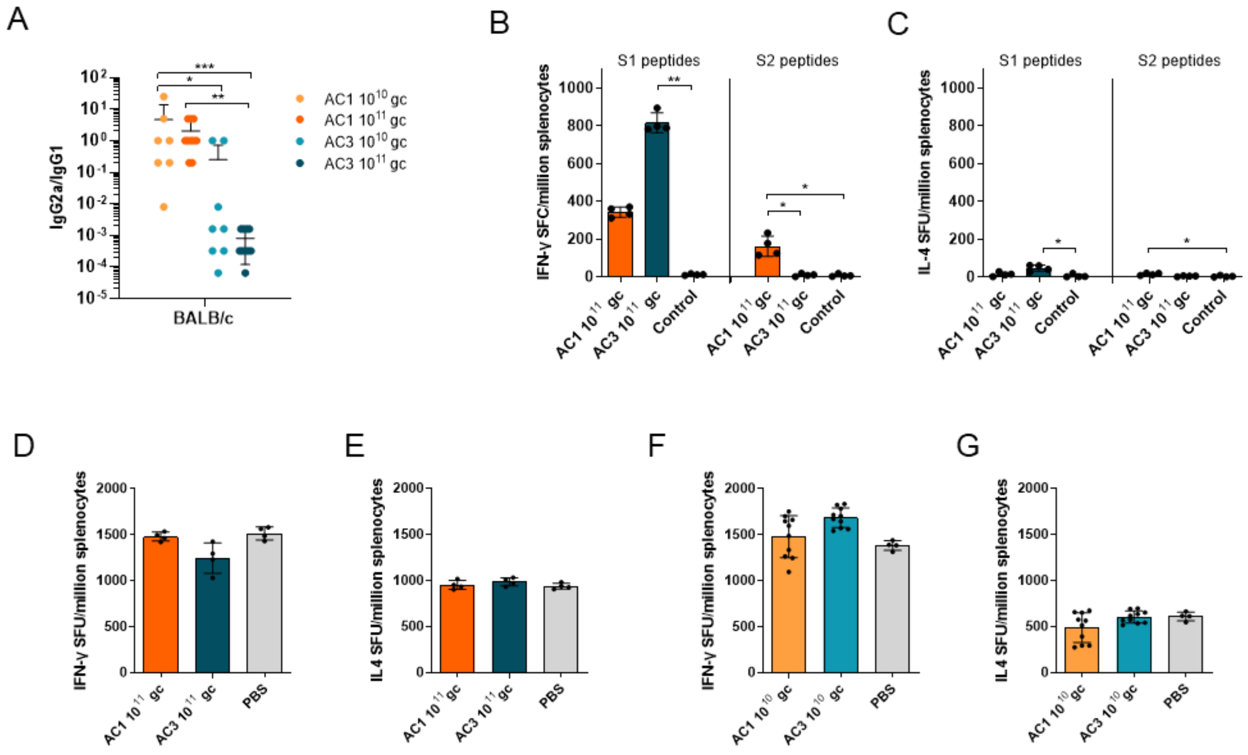

Figure S3. Related to Figure 2. Quality of the host response to AAVCOVID.
(A) Ratio of RBD-binding IgG2a and IgG1 antibody titers in serum samples harvested 4 weeks after vaccination of BALB/c mice 4 weeks after vaccination with 10^{11} gc of AC1 or AC3.
(B-C) Spot forming units (SFU) detected by IFN-γ (B) or IL-4 (C) ELISPOT in splenocytes extracted from BALB/c animals 4 weeks after vaccination with 10^{11} gc of AC1 or AC3 and stimulated with peptides spanning SARS-CoV-2 Spike protein for 48h.
(D-E) Spot forming units (SFU) detected by IFN-γ (D) or IL-4 (E) ELISPOT in splenocytes extracted from BALB/c animals 4 weeks after vaccination with 10^{11} gc of AC1 or AC3 and stimulated with 2 μg/ml concanavalin A (positive control) for 48h.
(F-G) Spot forming units (SFU) detected by IFN-γ (F) or IL-4 (G) ELISPOT in splenocytes extracted from C57BL/6 animals 6 weeks after vaccination with 10^{10} gc of AC1 or AC3 and stimulated with 2 μg/ml concanavalin A (positive control) for 48h.
For (A-G) data are represented as mean ± SD and groups were compared by Kruskal Wallis and Dunn's post-test.

Figure S4

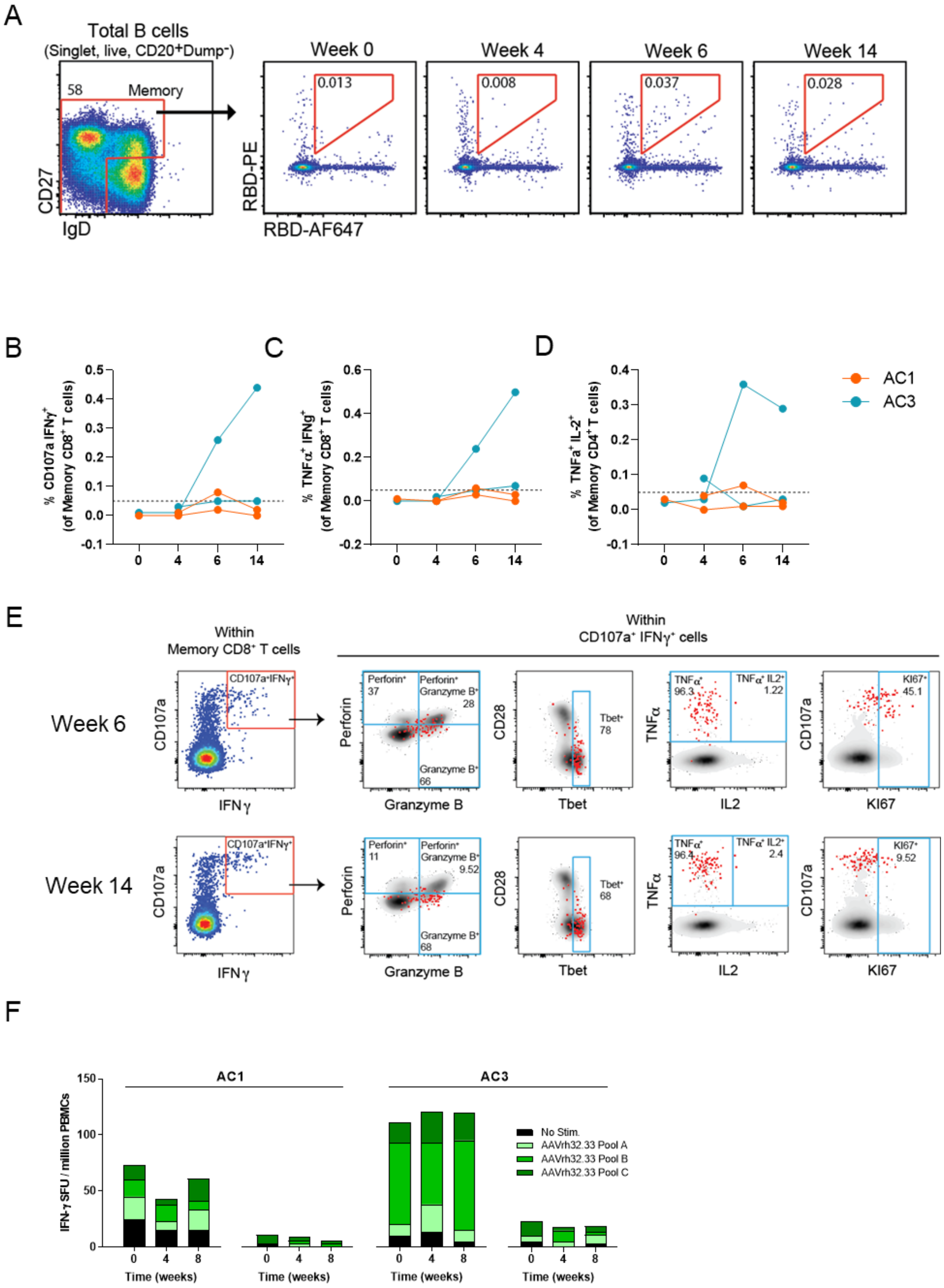
A
Total B cells
(Singlet, live, CD20+Dump-)
58
Memory
CD27
IgD
Week 0
Week 4
Week 6
Week 14
0.013
0.008
0.037
0.028
RBD-PE
RBD-AF647
B
C
D
% CD107a IFNγ+ (of Memory CD8+ T cells)
% TNFα+ IFNg+ (of Memory CD8+ T cells)
% TNFa+ IL-2+ (of Memory CD4+ T cells)
AC1
AC3
E
Within
Memory CD8+ T cells
Within
CD107a+ IFNγ+ cells
Week 6
Week 14
CD107a+IFNγ+
CD107a
IFNγ
Perforin
Granzyme B
CD28
Tbet
TNFα
IL2
KI67
F
AC1
AC3
IFN-γ SFU / million PBMCs
Time (weeks)
No Stim.
AAVrh32.33 Pool A
AAVrh32.33 Pool B
AAVrh32.33 Pool C

Figure S4. Related to Figure 3. Characterization of cellular immune responses in NHP.
(A) Identification of RBD-binding B cells with a memory phenotype (CD27+ or CD27-IgD-) in peripheral blood of a representative macaque at multiple dates post-vaccination.
(B-C) Background subtracted frequency of $CD107a^{+}IFN\gamma^{+}$ (B) or $TNF\alpha^{+}$ $IFN\gamma^{+}$ (C) memory $CD8^{+}$ cells responding to Spike peptides at baseline and at different time points after vaccination. The dotted line indicates the cutoff for positive responses.
(D) Frequency of background subtracted $TNF\alpha^{+}$ $IL2^{+}$ memory $CD4^{+}$ cells responding to Spike peptides at baseline and at different time points after vaccination. The dotted line indicates the cutoff for positive responses.
(E) Flow cytometry plots from AC3 female indicating the frequency of Perforin, Granzyme B, Tbet, TNFα, IL2 and KI67-positive cells within $CD107^{+}$ $IFN\gamma^{+}$ Memory $CD8^{+}$ T cells responding to Spike peptide pools at weeks 6 and 14 post vaccination. In the flow plots, total $CD107^{+}$ $IFN\gamma^{+}$ cells were depicted as red dots overlayed on total Memory $CD8^{+}$ T cells (black).
(F) Quantification of spot forming units (SFU) by ELISPOT in PBMC samples collected at different timepoints in animals treated with AC1 or AC3 and stimulated with peptides spanning AAVrh32.33 capsid sequence.

Figure S5

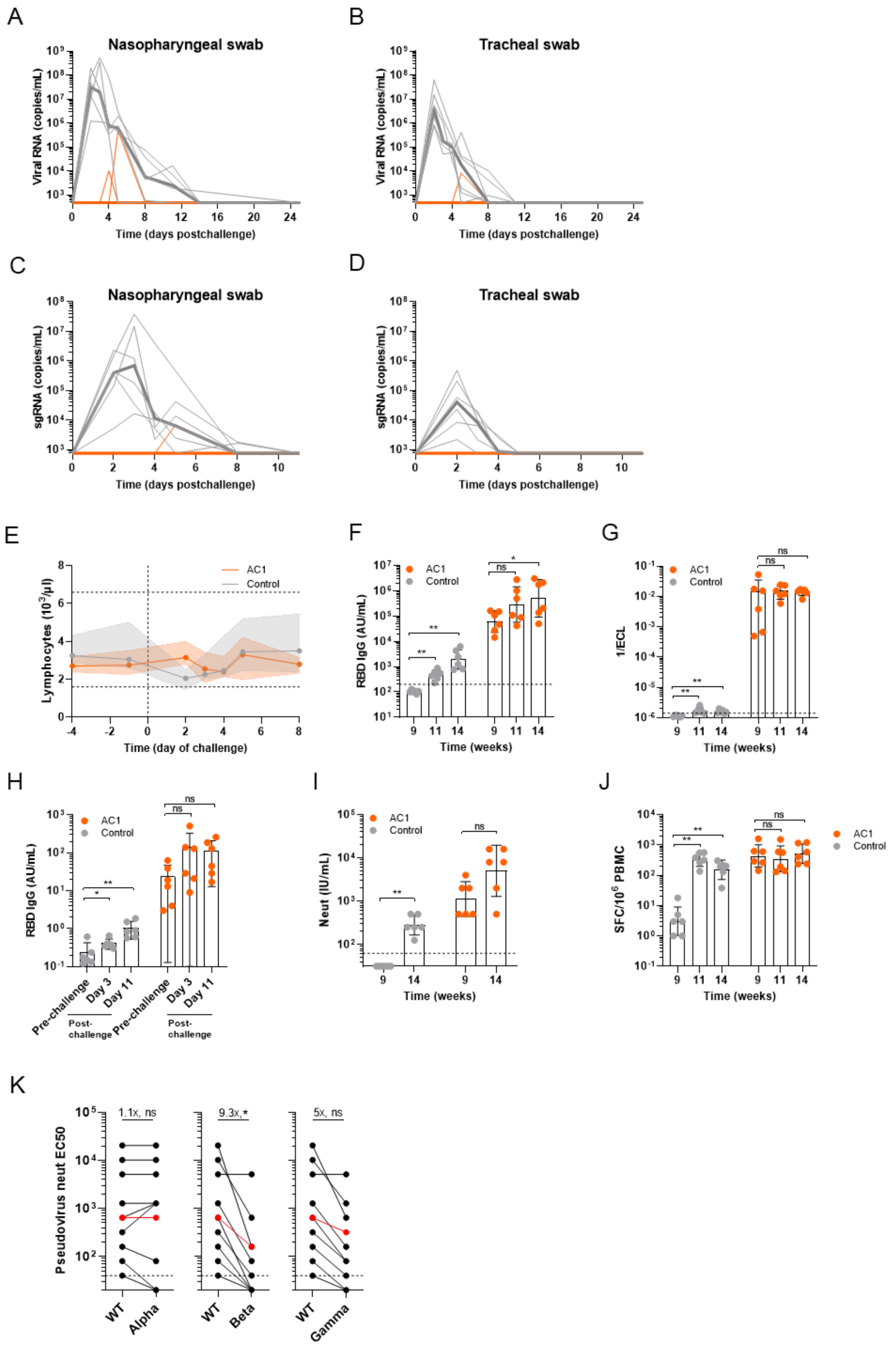

A
Nasopharyngeal swab
Viral RNA (copies/mL)
Time (days postchallenge)
B
Tracheal swab
Viral RNA (copies/mL)
Time (days postchallenge)
C
Nasopharyngeal swab
sgRNA (copies/mL)
Time (days postchallenge)
D
Tracheal swab
sgRNA (copies/mL)
Time (days postchallenge)
E
AC1
Control
Lymphocytes (10^3/µl)
Time (day of challenge)
F
AC1
Control
RBD IgG (AU/mL)
Time (weeks)
G
AC1
Control
1/ECL
Time (weeks)
H
AC1
Control
RBD IgG (AU/mL)
Pre-challenge
Day 3
Day 11
Post-challenge
I
AC1
Control
Neut (IU/mL)
Time (weeks)
J
AC1
Control
SFC/10^6 PBMC
Time (weeks)
K
Pseudovirus neut EC50
1.1x, ns
9.3x,*
5x, ns
WT
Alpha
Beta
Gamma

Figure S5. Related to Figure 4. Protection from SARS-CoV-2 challenge in Cynomolgus macaques vaccinated with AC1.

(A-B) SARS-CoV-2 viral RNA copies in nasopharyngeal (A) and tracheal swab (B) at several timepoints after 10^5 pfu SARS-CoV-2 challenge. Thin lines represent individual animals in each group and thick lines the median for each group (controls in grey and AC1-vaccinaed in orange).

(C-D) SARS-CoV-2 subgenomic RNA quantification (copies/mL) in copies in nasopharyngeal (C) and tracheal swab (D) at several timepoints after 10^5 pfu SARS-CoV-2 challenge. Thin lines represent individual animals in each group and thick lines the median for each group (controls in grey and AC1-vaccinaed in orange).

(E) Circulating lymphocyte count ($x10^3$/mL) before and during the first week after 10^5 pfu SARS-CoV-2 challenge.

(F-J) Immune responses in AC1-vaccinated and control animals after SARS-CoV-2 challenge. RBD-binding IgG in serum (F), ACE2-Spike binding inhibiting antibodies (G), RBD-binding IgG in BAL (H) pseudovirus neutralizing antibodies in serum (I) and cellular responses by ELISPOT (J) were measured. Geometric mean and geometric SD are represented. Mann Whitney tetst was used to compare between timepoints in each group.

(K) Neutralization of SARS-CoV-2 Alpha, Beta, Gamma and Delta variants of concern (VOC) by human convalescent plasma including NIBSC20/136 international reference (red dot and line) in a pseudovirus neutralization assay. Fold-change of geometric mean titers is displayed. Wilcoxon matched-pairs signed rank test was used to compare titers to the WT and VOC. The dotted line indicates the lower detection limit of the assay.

Figure S6

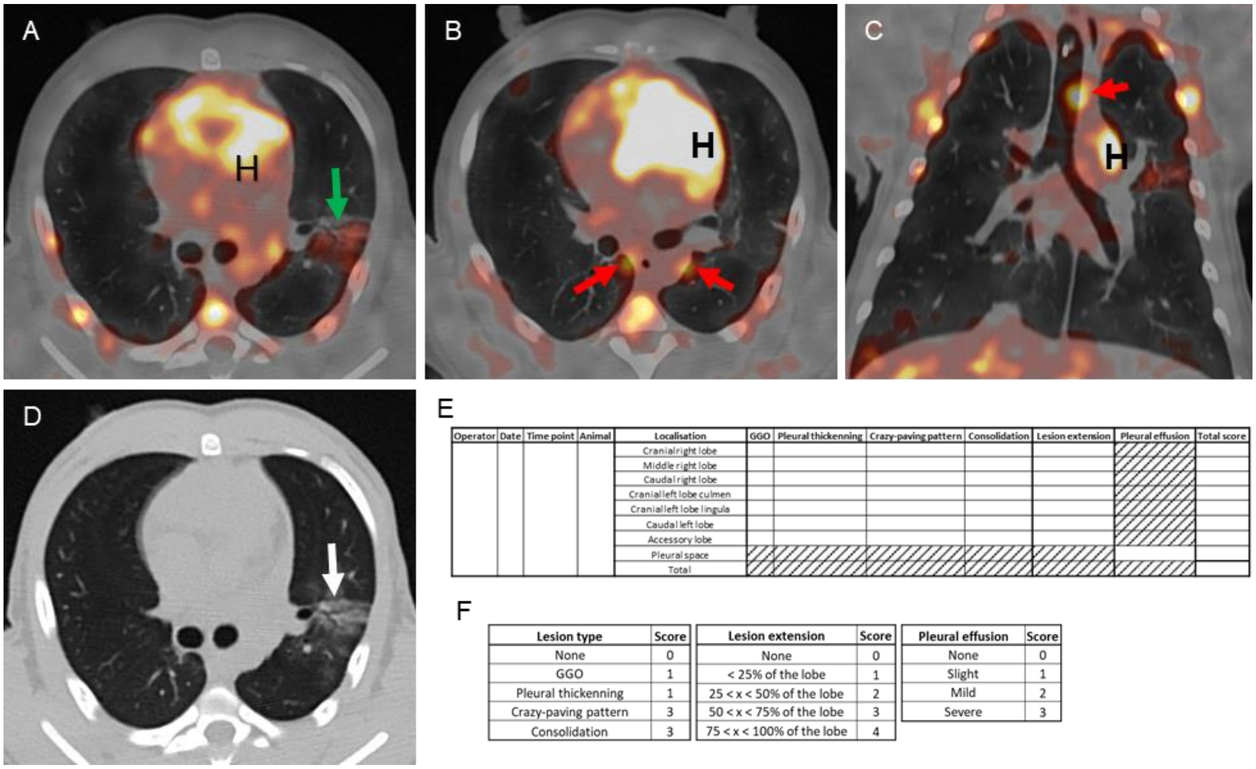

Operator	Date	Time point	Animal	Localisation	GGO	Pleural thickenning	Crazy-paving pattern	Consolidation	Lesion extension	Pleural effusion	Total score
				Cranial right lobe							
				Middle right lobe							
				Caudal right lobe							
				Cranial left lobe culmen							
				Cranial left lobe lingula							
				Caudal left lobe							
				Accessory lobe							
				Pleural space							
				Total							

Lesion type	Score
None	0
GGO	1
Pleural thickenning	1
Crazy-paving pattern	3
Consolidation	3

Lesion extension	Score
None	0
< 25% of the lobe	1
25 < x < 50% of the lobe	2
50 < x < 75% of the lobe	3
75 < x < 100% of the lobe	4

Pleural effusion	Score
None	0
Slight	1
Mild	2
Severe	3

Figure S6. Related to Figure 4. Illustrative images of [^{18}F]-FDG PET-CT and CT acquisitions in SARS-CoV-2 exposed unvaccinated cynomolgus macaque.
(A-C) Transversal and frontal PET (thermal signal) and CT merged slices acquired at day 5 post SARS-CoV-2 exposure. Parenchymal lung FDG hyperfixation (A, green arrow) can be detected in lesional areas (D, white arrow). Lung-draining lymph node FDG hyperfixation (B-C, red arrows) was also detected in these animals. Typical Ground Glass Opacities lesions were observed in lungs by CT (D, white arrow) and quantified using reporting tables and scoring grids (E-F). H= Heart
